# Hierarchical Nanostructures Self-Assembled from a Mixture System Containing Rod-Coil Block Copolymers and Rigid Homopolymers

**DOI:** 10.1038/srep10137

**Published:** 2015-05-12

**Authors:** Yongliang Li, Tao Jiang, Shaoliang Lin, Jiaping Lin, Chunhua Cai, Xingyu Zhu

**Affiliations:** 1 Shanghai Key Laboratory of Advanced Polymeric Materials, State Key Laboratory of Bioreactor Engineering, Key Laboratory for Ultrafine Materials of Ministry of Education, School of Materials Science and Engineering, East China University of Science and Technology, Shanghai 200237, China

## Abstract

Self-assembly behavior of a mixture system containing rod-coil block copolymers and rigid homopolymers was investigated by using Brownian dynamics simulations. The morphologies of formed hierarchical self-assemblies were found to be dependent on the Lennard-Jones (LJ) interaction *ε*_RR_ between rod blocks, lengths of rod and coil blocks in copolymer, and mixture ratio of block copolymers to homopolymers. As the *ε*_RR_ value decreases, the self-assembled structures of mixtures are transformed from an abacus-like structure to a helical structure, to a plain fiber, and finally are broken into unimers. The order parameter of rod blocks was calculated to confirm the structure transition. Through varying the length of rod and coil blocks, the regions of thermodynamic stability of abacus, helix, plain fiber, and unimers were mapped. Moreover, it was discovered that two levels of rod block ordering exist in the helices. The block copolymers are helically wrapped on the homopolymer bundles to form helical string, while the rod blocks are twistingly packed inside the string. In addition, the simulation results are in good agreement with experimental observations. The present work reveals the mechanism behind the formation of helical (experimentally super-helical) structures and may provide useful information for design and preparation of the complex structures.

Amphiphilic block copolymers have a capability to self-assemble into ordered nanostructures in selective solvents, such as spherical micelles, cylindrical micelles, and vesicles^1^,^2^,^3^,^4^. These nanostructures have attracted considerable interests due to their wide applications in drug delivery, coating, cosmetics, nanoreactors, and so on^5^,^6^. So far number of researches have been directed toward the self-assembly of flexible block copolymers. In contrast with the flexible block copolymers, block copolymers containing rigid blocks can organize into more well-ordered nanostructures, since the rigid segments can lead to orientation organization^7^,^8^. The self-assembly mechanism is relatively complicated for rod-coil block copolymers in selective solvents and it is a challenging task to understand exactly how the observed structures were formed.

Blending a second component with the block copolymer systems is an efficient approach to manipulate the aggregate structures relative to the design of a new copolymer with novel molecular structures^9^. The second component may be a block copolymer or homopolymer. The aggregate structures can be easily tuned by variation of properties of the mixture system such as the mixture ratio and interaction parameters. The cooperative self-assembly of two-component polymers has received much attentions in experimental studies^10^,^11^,^12^,^13^,^14^,^15^,^16^,^17^,^18^,^19^,^20^,^21^, because of the more opportunities for creating novel nanostructures. For example, Hayward *et al.* reported multicompartment micelles self-assembled from polystyrene-*b*-poly(ethylene oxide) (PS-*b*-PEO) diblock copolymers by introducing hydrophobic PS-*b*-poly(vinylpyridine) (PS-*b*-PVP) or PS-*b*-polyisoprene (PS-*b*-PI) copolymers^10^. Wormlike micelles with microphase-separated spherical or cylindrical domains of PVP (or PI) block were observed, depending on the mixture ratio. Compared to the mixture consisting of full flexible polymers, when one or both of the two components contain rigid segments, the liquid crystal effects of rigid segments could be expected to greatly affect the self-assembly behaviors.

For two-component polymers with rigid segments, the polypeptide-based copolymers are promising models for investigating the self-assembly behavior^22^,^23^,^24^,^25^,^26^,^27^,^28^,^29^. The polypeptide copolymers exhibit significant advantages in controlling both the structures and functions of the supramolecular aggregates^30^,^31^,^32^. In our previous work, we studied the self-assembly behavior of mixture systems containing poly(*γ*-benzyl-L-glutamate)-*b*-poly(ethylene glycol) (PBLG-*b*-PEG) block copolymers and PBLG-*g*-PEG graft copolymers^22^. The block copolymers can form spherical micelles or vesicles, while the graft copolymers can self-assemble into vesicles. When the two copolymers are mixed, cylindrical hybrid micelles are observed. In addition, we have carried out a preliminary study on the self-assembly behavior of PBLG-*b*-PEG/PBLG mixtures in aqueous solution.^25^ The experiments revealed that the PBLG homopolymers packed side-by-side to form bundles, and the PBLG-*b*-PEG block copolymers wrapped on the homopolymer bundles. Supramolecular structures such as abacus-like (beads-on-wire) structures, super-helices, and plain fibers can be sequentially observed as the self-assembling temperature decreases. However, the detailed structure and important information of these hierarchical aggregates cannot be provided through the experimental method, such as the chain ordering and twisting information. Moreover, the formation mechanism of these superstructures should be further verified.

Apart from the experimental investigations, theory and computer simulations have emerged as powerful tools to study the self-assembly behavior of two-component polymers^33^,^34^,^35^,^36^,^37^,^38^,^39^,^40^. They can provide more straightforward results than pure experiments, and overcome the limitation inherent in experiments. So far, various approaches, such as self-consistent field theory (SCFT)^33^,^34^,^35^,^36^, molecular dynamics (MD) simulations^37^, and dissipative particle dynamics (DPD) simulations^38^, have been successfully employed to investigate the aggregation behavior of polymer mixtures in solution. For example, Xu and coworkers used the SCFT method to investigate the self-assembly of homopolymer and diblock copolymer mixtures in selective solvents^35^. By varying the mixture ratio and homopolymer chain length, various morphologies including vesicles, circle- and line-like micelles, and their mixtures were predicted. Zhong *et al.* simulated the micellization of linear and star ABC triblock copolymer mixtures^38^. The structures of multicompartment micelles were found to be governed by mixture ratio and copolymer composition. So far, the publications on the two-component polymers are mainly focused on flexible polymers, and the theoretical and simulation studies on the mixtures with rod-coil copolymers or rigid homopolymers are very limited. Brownian dynamics (BD), as a solvent-free molecular dynamics method, presents more predominant in simulating rod-coil copolymers and computation efficiency^41^,^42^,^43^. We have applied the BD method to the study of micelle structures of rod-coil diblock copolymers. Twisting string and disk micelles were observed^41^. Such success of the BD simulation made it to be successfully extended to examine the self-assembly behavior of rod-coil block copolymer/rigid homopolymer mixtures^25^. The preliminary simulations well reproduced the general features of the self-assemblies from such a mixture system. However, many important issues remain unsolved in such complex systems, and the mixture systems need to be explored further.

In the present work, we performed a Brownian dynamics simulation to study the self-assembly behavior of mixture systems consisting of rod-coil block copolymers and rigid homopolymers in dilute solution. The effects of interaction parameter *ε*_RR_ between rod blocks, block length, and mixture ratio on the aggregate structures were examined, and various structures including abacus-like, helical structures, plain fiber, and unimers were obtained. The thermodynamic stability regions of these structures in space of *ε*_RR_ and block length were constructed. The ordering information of rod blocks and twisting information in the helical structures were also provided to reveal the formation mechanism of these complex structures.

## Results

**Figure 1:**
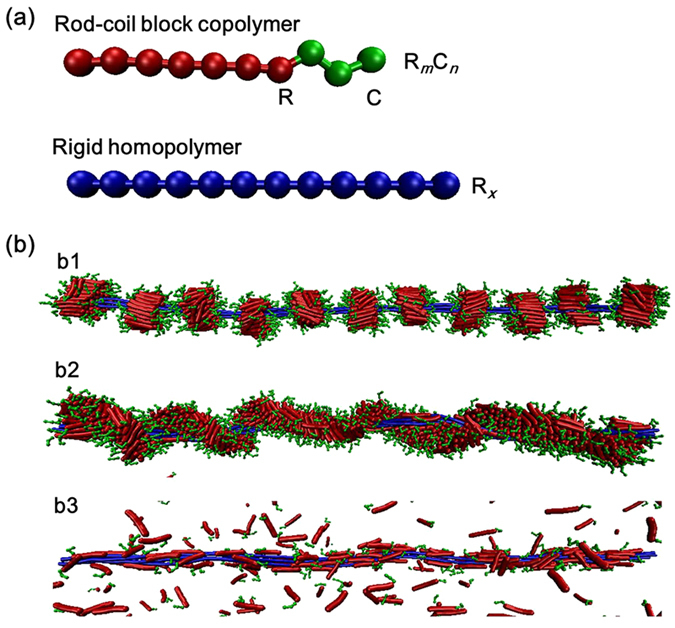
(**a**) Coarse-grained model of a rod-coil block copolymer and a rigid homopolymer. The block copolymer and homopolymer are denoted by the type of **R**_*m*_**C**_*n*_ and **R**_*x*_. The beads colored by red, green, and blue represent rigid **R** block, flexible **C** block, and rigid **R** homopolymer chain, respectively. (**b**) Simulated morphologies self-assembled from **R**_7_**C**_3_/**R**_150_ mixtures at various *ε*_RR_: (b1) *ε*_RR_ = 2.5, (b2) *ε*_RR_ = 2.1, and (b3) *ε*_RR_ = 1.6.

Very recently, we have discovered that a mixture of rod-coil block copolymers and rigid homopolymers was able to co-assemble into supramolecular structures such as super-helices. The preliminary results suggest that a number of parameters, including the interactions between rods, mixture ratio, and chain length, *etc*., play important roles in determining the aggregate structures of the mixtures. It is therefore worth to carry out further studies in order to understand the underlying mechanism of the cooperative self-assembly. In this work, self-assembly of rod-coil block copolymer/rigid homopolymer mixtures was studied, and the effect of interactions between rods, chain length, and mixture ratio was examined. A coarse-grained model of **R**_*m*_**C**_*n*_/**R**_*x*_ mixture was constructed as typically shown in Fig. 1a. The rod block length *L*_R_ and coil block length *L*_C_ in block copolymer are denoted by the bead numbers of **R** and **C** blocks in one copolymer chain, respectively (*i.e.*, *L*_R_ = *m*, *L*_C_ = *n*). The mixture ratio *φ* of block copolymer to homopolymer is defined as the chain number ratio of the block copolymers to homopolymers. The simulation details are presented in Methods section. In what follows, the effects of these parameters were examined. In all simulations, the homopolymer chain number was fixed as 4, and the **R** bead number in one homopolymer chain was set to be 150. The chain number of **R**_*m*_**C**_*n*_ block copolymers was set as 660, except for the study of the dependence of aggregate structures on mixture ratio *φ*.

### Effect of Interaction Strength between Rod Blocks on Aggregate Structure

As revealed by previous communication^25^, the interactions between rods (*ε*_RR_), which can be related to temperature in experiments, is an important factor determining the self-assembled structures of rod-coil block copolymer/rigid homopolymer. In this sub-section, the effect of such interactions was examined. The mixtures contain 660 **R**_7_**C**_3_ copolymers and 4 **R**_150_ homopolymers. The total bead number is 7200, therefore the number density of the system is roughly 0.0021. The *ε*_RR_ was varied from 2.7 to 1.3. The larger the value of *ε*_RR_, the more hydrophobic the **R** blocks.

Figure 1b presents the effect of *ε*_RR_ on the simulation results. When *ε*_RR_ is higher (*ε*_RR_ ≥ 2.5), the **R**_7_**C**_3_/**R**_150_ mixtures self-assemble into an abacus-like structure (beads-on-wire), in which **R**_7_**C**_3_ block copolymers aggregate into separating disks around **R**_150_ bundles (Fig. 1b1). Inside the abacus-like structure, the homopolymer rods are packed closely forming the inner axis. We can also see the orientation packing of rod blocks in each disk. As *ε*_RR_ decreases to the range between 2.4 and 2.1, the **R**_7_**C**_3_ block copolymers become helically wrapped on the **R**_150_ bundles, as shown in Fig. 1b2. The block copolymers continuously form a helical string along the major axis of homopolymer bundles, in which rod blocks are also aligned twistingly with each other. With further decreasing *ε*_RR_, the block copolymers cannot enwrap fully the homopolymer bundles, as seen from the snapshot of *ε*_RR_ = 1.6 in Fig. 1b3. The **R**_150_ bundles are randomly coated with **R**_7_**C**_3_ copolymers, but many **R**_7_**C**_3_ chains are freely dispersed in the simulation cell. The aggregate is no longer a helical fiber but a plain fiber. Finally, all unimers come out from the fiber, and no aggregates are formed (snapshot is not included).

**Figure 2:**
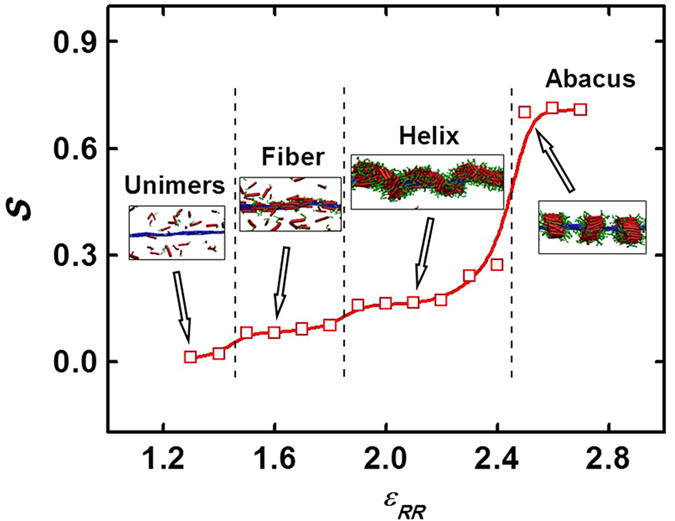
Order parameter *S* of **R**_7_ blocks in the aggregates formed by **R**_7_**C**_3_/**R**_150_ mixtures as a function of *ε*_RR_. The insert shows the typical morphology snapshots of aggregates at various *ε*_RR_. The regions of different structures are separated by dashed lines.

From Fig. 1b, we learn that a structure transition from abacus to helix, to plain fiber, and finally to unimers appears for **R**_7_**C**_3_/**R**_150_ mixtures with decreasing interaction strength *ε*_RR_ between rod blocks. To identify the structural transition of aggregates and molecular packing of rod blocks, the order parameter *S* of **R**_7_ blocks in the aggregates was calculated. The *S* as a function of *ε*_RR_ is shown in Fig. 2. The insert shows the typical morphology snapshots of aggregates at various *ε*_RR_. As can be seen, *S* is larger than 0.7 in the abacus region when *ε*_RR_ ≥ 2.5 and then sharply decreases to about 0.2 with decreasing *ε*_RR_. The higher *S* value indicates that the rod blocks are orientated and packed regularly in the copolymer disks. The sharp decrease of *S* is ascribed to the abacus-helix transition. In the helix region, the **R**_7_ blocks are twistingly packed in the helical string, therefore *S* decreases dramatically. With further decreasing *ε*_RR_, the *S* is very low, implying the packing of rod blocks is disordered in the fiber and unimer regions.

**Figure 3:**
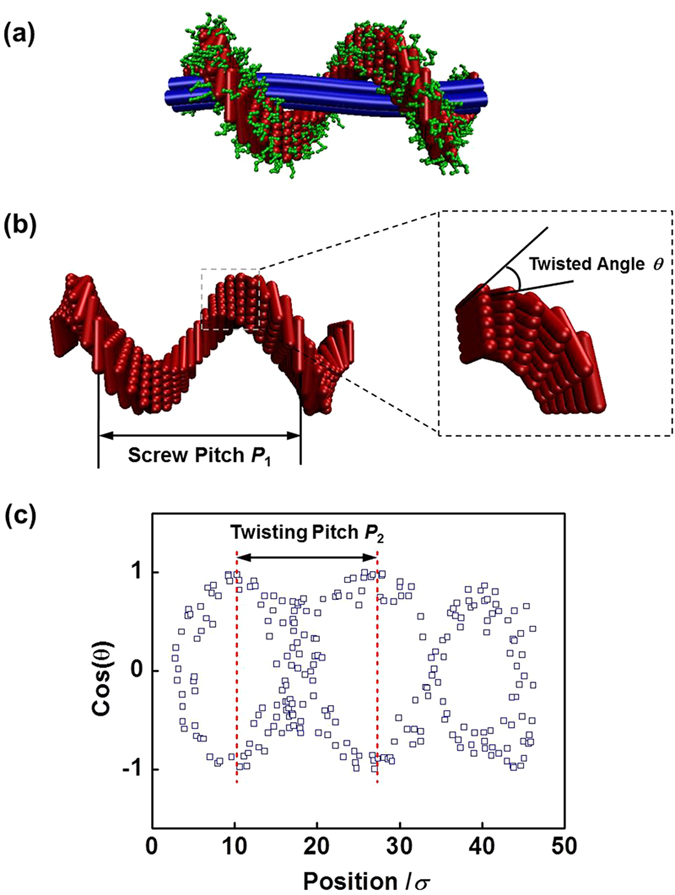
(**a**-**b**) Schematic representation for the helical structure and domain of rod blocks of block copolymers. In figure (**b**), the enlarged view indicates the local twisting packing of rod blocks. The sketch of definitions of screw pitch *P*_1_ and twisted angle *θ* is also shown. (**c**) Typical result of cos(*θ*) as a function of position along the long axis in the helical fiber of **R**_7_**C**_3_/**R**_150_ mixtures at *ε*_RR_ = 2.1. The distance between dashed lines was defined as the local twisting pitch *P*_2_ of rod blocks.

As mentioned above, in the self-assembly of mixture, the block copolymers are wrapped on the surface of homopolymer bundles to form structures such as helical string. To illustrate the structure of helical string more clearly, we show schematic representations of the helical structure and the domain of rod blocks of block copolymers in Fig. 3a–b. It can be seen that the homopolymer bundles form the inner axis and block copolymers form the screw through ordered packing of the rod blocks. The rod blocks tend to align in an orientation vector, and such a vector is gradually changed along the axis of the bundle, which can be seen in the enlarged view of local packing of rod blocks (Fig. 3b). The global helical distribution of block copolymers is in a large-length-scale, while the local twisting packing of rod blocks is in a small-length-scale. The interplay of these two scale orderings determines the final hierarchical structures. In addition, two structural parameters of helix, *i.e.*, screw pitch and twisted angle, were defined, and the sketch of their definitions is shown in Fig. 3b. The screw pitch *P*_1_ of helix is the length along the long axis of the helix string in a helix period, and the twisted angle *θ* is the angle between each rod block and orientation direction. Figure 3c shows a typical result of cos(*θ*) as a function of position along the long axis in the helical string of **R**_7_**C**_3_/**R**_150_ mixtures at *ε*_RR_ = 2.1. The absolute value of cos(*θ*) gradually increases to 1.0 and then decreases to 0.0 again. The distance along the axis in one periodic change of cos(*θ*) (distance between dashed lines) was defined as the local twisting pitch *P*_2_ of rod blocks, which is about 17.0*σ* for this case of *ε*_RR_ = 2.1.

The helical string with double-twisting structure is an interesting finding in the present work. For pure rod-coil block copolymers, we have observed a twisting string in our previous study^41^, which is a single-twisting structure (no global helix). The double-twisting structure formed by introducing long rigid homopolymers was found for the first time. The homopolymer bundles provide a surface for block copolymers to assemble on, and at certain interaction strength the block copolymers form a helical string wrapping the homopolymer bundles. The local twisting packing of rod blocks is analogous to the packing manner of rod blocks in a string micelle formed by pure rod-coil block copolymers. They are both examples of twisting structures formed by nonchiral polymers, which provide new strategies for preparing helical superstructures. However, in rod-coil block copolymer systems, the string micelle could split into smaller string micelles when the number of copolymers is large enough^41^. Compared to that, in the rod-coil block copolymer/rigid homopolymer mixture systems, the helical string can be elongated under the directing of homopolymer bundles regardless of the number of copolymers.

### Effect of Block Length on Aggregate Structure

The block length of copolymers is another important parameter determining the cooperative self-assembly behaviors. We then examined the effect of rod block lengths *L*_R_ and coil block lengths *L*_C_ on the structures of self-assembled aggregate. The simulations were carried out for two cases. One is that the *L*_R_ was varied from 6 to 9, while the *L*_C_ was fixed as 3. The other scenario is that the *L*_C_ was changed from 3 to 6, while the *L*_R_ kept unchanged (*L*_R_ = 7). Combing the effect of interaction strength *ε*_RR_, the thermodynamic stability regions of aggregate structures for various block lengths were constructed.

**Figure 4:**
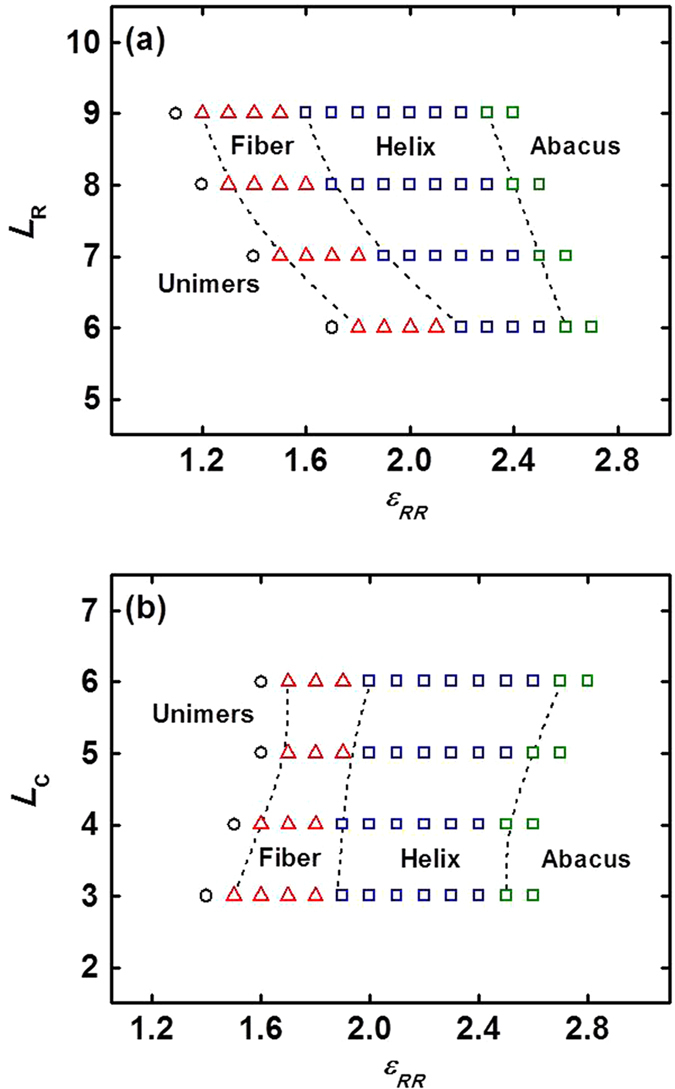
Morphology stability regions of rod-coil block copolymer/rigid homopolymer mixtures in space of: (**a**) *L*_R_ vs *ε*_RR_ and (**b**) *L*_C_ vs *ε*_RR_. Regions of abacus, helix, plane fiber, and unimers are shown. In figure (**a**), the *L*_C_ is fixed as 3, while in figure (**b**), the *L*_R_ is 7.

Figure 4a,b shows the morphology stability regions in space of *L*_R_ vs *ε*_RR_ and *L*_C_ vs *ε*_RR_, respectively. The morphology includes abacus, helix, plain fiber, and unimers. As shown in Fig. 4a, the rod-coil block copolymer/rigid homopolymer mixtures (*e.g.*, **R**_7_C_3_/**R**_150_ mixtures) form an abacus-like structure at higher *ε*_RR_. As the *ε*_RR_ decreases, a transition from abacus-like structure to helical structure and then to plain fiber was shown. With further decreasing *ε*_RR_, the system gets into the unimer region. On the other hand, with increasing the rod block length *L*_R_, the region of helix becomes wider, while the region width of plain fiber keeps unchanged roughly. The boundaries of these regions tend to shift toward lower *ε*_RR_. It suggests that at a constant *ε*_RR_ the abacus-like structure is easier to form than the helical structure and plain fiber for longer rod block. Figure 4b shows the region widths of helix and plain fiber have a slight change, while the region boundaries move to higher *ε*_RR_ as the coil block length *L*_C_ increases, which is opposite to the effect of *L*_R_. The mixtures tend to form the abacus-like structure for shorter coil block but form the helical structure or plain fiber for longer coil block at a constant *ε*_RR_.

**Figure 5:**
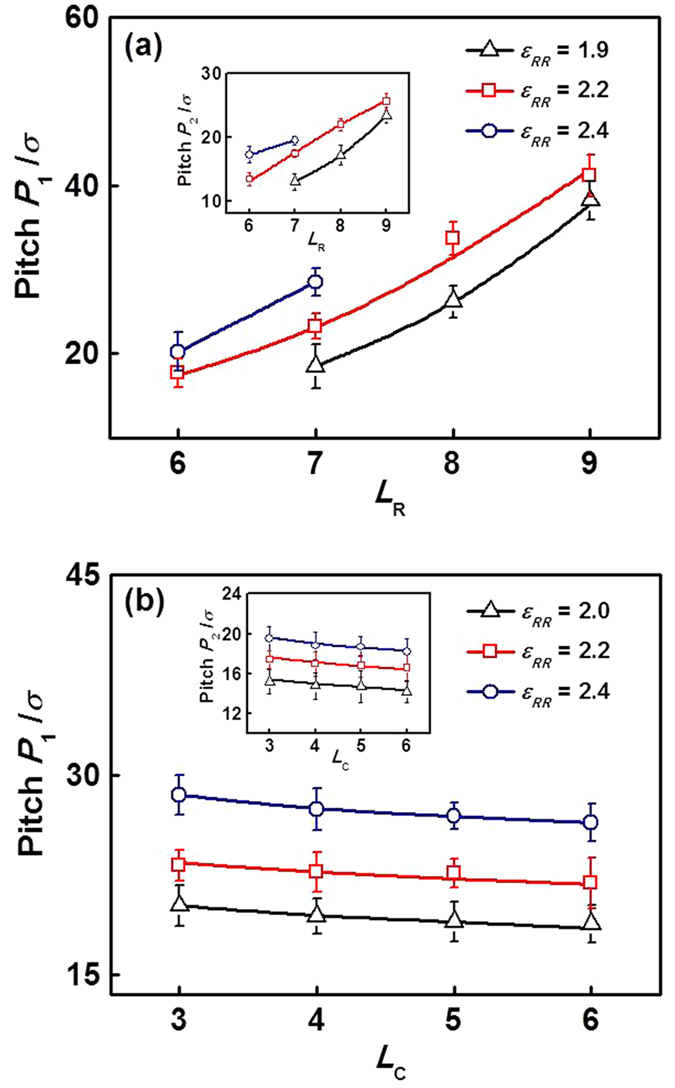
(**a**) Screw pitches *P*_1_ of helical structures formed by **R**_*m*_**C**_3_/**R**_150_ mixtures as a function of *L*_R_ at *ε*_RR_ = 1.9, 2.2, and 2.4. The insert shows the local twisting pitches *P*_2_ of rod blocks as a function of *L*_R_ at various *ε*_RR_. (**b**) Pitches *P*_1_ of helical structures formed by **R**_7_**C**_*n*_/**R**_150_ mixtures with various *L*_C_ at *ε*_RR_ = 2.0, 2.2, and 2.4. The insert shows the *P*_2_ *versus L*_C_ at various *ε*_RR_.

From the simulation results, we learn that the lengths of both rod and coil blocks affect the structural transition of aggregates. In fact, for a given aggregate structure, the block length may also affect the structure details of the aggregates. For example, the helical structures which occupy the major part of the morphology stability regions may possess different pitches at various block lengths. We then examined the screw pitches *P*_1_ of helical structures and local twisting pitch *P*_2_ of rod blocks as a function of *L*_R_ and *L*_C_. The results are presented in Fig. 5. Figure 5a presents the pitches *P*_1_ of helical structures with various *L*_R_ at *ε*_RR_ = 1.9, 2.2, and 2.4. The insert shows the *P*_2_ as a function of *L*_R_ at various *ε*_RR_. These two pitches were both found to increase markedly with increasing *L*_R_ at a fixed *ε*_RR_. For example, at *ε*_RR_ = 2.2, for *L*_R_ = 6 the pitch *P*_1_ is 17.7*σ* (*σ* is the diameter of LJ bead, *P*_2_ = 13.4*σ*), while for *L*_R_ = 9 the *P*_1_ is up to 41.2*σ* (*P*_2_ = 25.6*σ*). These results indicate that the helical wrapping of block copolymers on homopolymer bundles is in a more extended manner for longer rod block, and the local twisting of rod blocks is easier to occur for shorter rod block. We can also view the effect of *ε*_RR_ on the screw pitch *P*_1_ and twisting pitch *P*_2_. With decreasing *ε*_RR_, the two pitches both decrease. It suggests that the block copolymers are easier to be helically distributed and rod blocks are easier to twistingly pack at lower *ε*_RR_. Figure 5b shows the screw pitches *P*_1_ of helical structures as a function of *L*_C_ at *ε*_RR_ = 2.0, 2.2, and 2.4. The insert shows the twisting pitch *P*_2_ *versus L*_C_ at various *ε*_RR_. As can be seen, *P*_1_ and *P*_2_ decrease slightly with *L*_C_ increases, while the pitches increase markedly with increasing *ε*_RR_. The structure diversity of helices for various *L*_C_ is not obvious relative to the helices formed by mixtures with different *L*_R_.

### Effect of Mixture Ratio on Aggregate Structure

The effect of mixture ratio *φ* (block copolymer/ homopolymer) on the aggregate structures was further examined. We mainly focused on the helical structure and explored the relationship between the structure of helices and mixture ratio. The interaction parameter *ε*_RR_ was chosen to ensure the formation of helical structures. The chain number of homopolymer rods kept constant, while the chain number of **R**_7_**C**_3_ block copolymers was varied to realize various mixture ratios.

**Figure 6:**
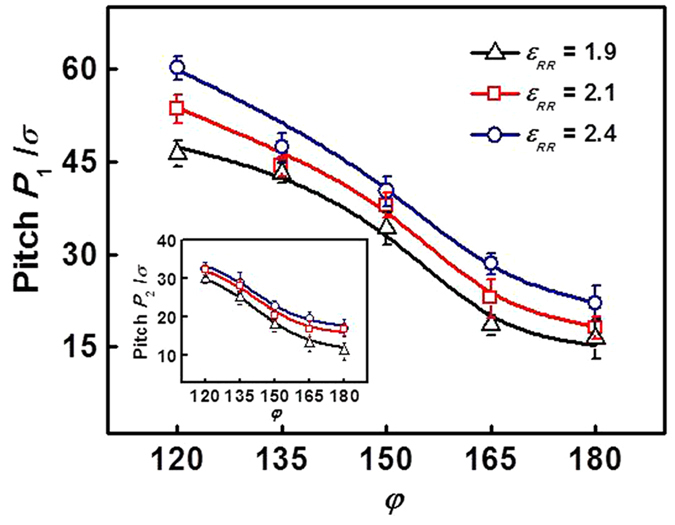
Screw pitches *P*_1_ of helices formed by **R**_7_**C**_3_/**R**_150_ mixtures with various mixture ratios *φ*. The interaction strength of rod blocks is *ε*_RR_ = 1.9, 2.1, or 2.4. The insert shows the local twisting pitches *P*_2_ of rod blocks as a function of *φ* at various *ε*_RR_.

Figure 6 shows the screw pitches *P*_1_ of helices formed by mixtures with various *φ* at *ε*_RR_ = 1.9, 2.1, and 2.4. The insert shows the local twisting pitches *P*_2_ of rod blocks as a function of *φ* at various *ε*_RR_. The value of *φ* was changed from 120 to 180, as the chain number of block copolymers was set in the range of 480 and 720. From the figure, we can see both the effects of *φ* and *ε*_RR_. For any *ε*_RR_, the pitches *P*_1_ and *P*_2_ decrease rapidly with increasing *φ* from 120 to 180. At a fixed *φ*, two pitches have an increase with the increase of *ε*_RR_. When the value of *φ* is smaller, the helical wrapping of block copolymers on homopolymer bundles is more extended (*P*_1_ is larger), and the rod blocks take on a smaller twisting degree (*P*_2_ is larger). As the *φ* increases, the helical wrapping of copolymers becomes more sufficient (the pitch *P*_1_ becomes smaller) and the contact between the homopolymer surface and solvents decreases. The decrease of screw pitch *P*_1_ could be ascribed to the increase in chain number of block copolymers. The helical string on homopolymer bundle surface becomes longer, but the homopolymer bundles keep a fixed length. To wrap the homopolymer bundle surface effectively, the block copolymers have to be packed in a relatively shrinking manner. The closer packing of rod blocks also leads to the decrease of twisting pitch *P*_2_ of rod blocks. Figure 6 indicates that the mixture ratio has a prominent effect on the screw pitches of formed helical structures and local twisting pitches of rod blocks. However, only the results of systems with larger mixture ratio were presented here. The effect of mixture ratio on the aggregate structures at smaller *φ* is revealed in the next section, through a comparison with the experimental results.

### Comparison with Experimental Observations

In previous work^24^,^25^, we reported a study of cooperative self-assembly of a mixture of PBLG-*b*-PEG block copolymers and PBLG homopolymers. The PBLG blocks take rigid α-helix conformation, while the PEG are flexible chains. They can correspond to the rod and coil blocks in the simulations. We have examined the effect of self-assembling temperature on the aggregate structures. The results show that the PBLG_141_-*b*-PEG_112_/PBLG_2411_ binary system (the subscripts denote the polymerization degree (DP) for each segment) self-assemble into abacus-like structures at 40 °C. With decreasing the temperature to 20 °C, the super-helical structures were observed. Further decreasing the temperature, plain fibers were formed at 5 °C. The experiments indicated that the self-assembling temperature has a prominent influence on the aggregate structures.

In the experiments, the solubility of PEG decreases with increasing temperature^44^, which means that the interaction between solvent (water) and hydrophilic PEG segments is weaker at higher temperature. In this case, the PEG block becomes more hydrophobic and PBLG-*b*-PEG copolymers have stronger tendency to aggregate at higher temperature. Since there are no solvents in Brownian dynamics simulation, the strong aggregation tendency of the PBLG-*b*-PEG copolymers was realized by tuning the parameter of *ε*_RR_^25^. A decrease of interaction strength *ε*_RR_ between the **R** blocks corresponds to a decrease of temperature in the experiments.

In the simulations, as shown in Fig. 1b, the **R**_7_**C**_3_/**R**_150_ mixtures self-assemble into an abacus-like structure with **R**_7_**C**_3_ disks around **R**_150_ bundles at *ε*_RR_ = 2.4, where the parameter settings are according to the experiments. In the experiments, the number average molecular weight (*M*_n_) of PBLG homopolymer is 528000 (DP = 2411), and the *M*_n_ for typical PBLG-*b*-PEG is 36000 (PBLG: *M*_n_ = 31000, DP = 141; and PEG: *M*_n_ = 5000, DP = 112). Therefore, the ratio of PBLG to PBLG-*b*-PEG (in terms of *M*_n_) is nearly 15. In the simulations, the number ratio of rigid homopolymer to copolymer model is set to be 15, where the coarse-grained homopolymer contains 150 beads and the copolymer model contains 10 beads. Meanwhile, the ratio of molecular weight *M*_n_ of PBLG homopolymer to that of PBLG block of PBLG-*b*-PEG is about 17, and the setting ratio of R_150_ homopolymer to R_7_ block in the simulation model is close to this value.

In addition, the model of block copolymer was chosen such that the bulk density of pure species^45^ or the relative lengths of the blocks^46^ matches the experimental data. In the simulations, the copolymer model was chosen by renormalizing both the bulk weight densities and block lengths. From the experiments, we learned that the *M*_n_ of PBLG block and PEG block in PBLG-*b*-PEG copolymers are 31000 and 5000, respectively. First, the number of BD beads was renormalized by keeping the bulk density identical in the simulations and experiments, and then a ratio of 31000 / 5000 was obtained. In this case, 3.6 beads for the PBLG block can form a 0.54 nm helix^46^, while 1 bead for the PEG block occupies 0.35 nm^47^. Second, the number of BD beads was renormalized by the length of rod block and coil block, and we obtained the relative number of beads for **R** and **C** blocks as (31000 × 0.54/3.6) : (5000 × 0.35/1) ≈ 7 : 2.63. As a result, the model of **R**_7_**C**_3_ rod-coil block copolymers (contains 7 **R** beads and 3 **C** beads) was adopted in the simulations, which can capture the essential feature of PBLG-*b*-PEG block copolymers in the experiments. As the *ε*_RR_ decreases, the aggregate morphology was transformed to a helical structure with **R**_7_**C**_3_ helically wrapping on **R**_150_ bundles (at *ε*_RR_ = 2.1) and a plain fiber with **R**_8_**C**_3_ randomly packing on **R**_150_ bundle surface (at *ε*_RR_ = 1.5). The transition from abacus to helix and then to plain fiber is in good accordance with the experimental observations.

**Figure 7:**
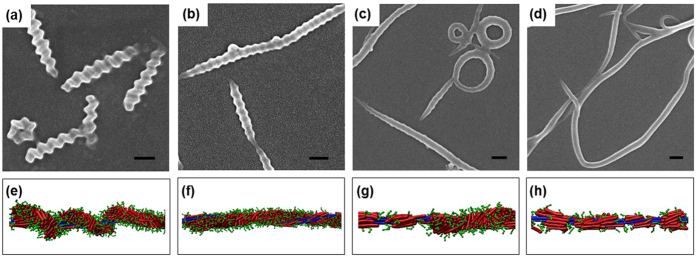
(**a**-**d**) SEM images of fibers prepared from the PBLG_141_-*b*-PEG_112_/PBLG_2411_ mixtures with various molar ratios of PBLG-*b*-PEG block copolymer to PBLG homopolymer: (**a**) 130, (**b**) 35, (**c**) 14, and (**d**) 6. Scale bars: 200 nm; temperature: 20 °C. (e-h) Fragments of simulated structures for **R**_7_**C**_3_/**R**_150_ mixtures with various mixture ratios *φ*: (**e**) *φ* = 165, (**f**) *φ* = 112.5, (**g**) *φ* = 80, and (**h**) *φ* = 52.5. The interaction parameter *ε*_RR_ is fixed as 2.1.

Here, we carried out further comparisons between the simulations and experiments. The experimental details are available in Supporting Information. In addition to temperature, the mixture ratio is another factor influencing the cooperative self-assembly. The experimental results, as shown in Fig. 7a-d, indicate that the helices are clearly visible when the molar ratio of PBLG-*b*-PEG block copolymer to PBLG homopolymer is larger (130). With decreasing the molar ratio, the helical structures tend to be less visible, and then fibers with no helical structure but a rough surface appear. Finally, completely plain fibers are obtained (for details, see sections 1 and 2 of Supporting Information). The simulations obtained similar results, as shown in Fig. 7e-h. The parameter setting of the simulation models corresponds to the essential feature of the experimental samples. For example, *φ* is changed from 165 to 52.5, according to the molar ratio of PBLG-*b*-PEG copolymer to PBLG homopolymer varying from 130 to 6 in experiments. As can be seen, with decreasing the mixture ratio *φ*, the regular helical structure is gradually transformed into fibers without regular helical structures. When the *φ* is small enough (52.5), the block copolymers simply covered the homopolymers, and plain fibers were formed. The block copolymers tend to protect the homopolymer surface from being exposed to the solvents, but the block copolymer chains cannot cover the homopolymer bundles sufficiently, and no helical structures are formed. We can find that a good qualitative agreement between simulations and experiments was shown again.

**Figure 8:**
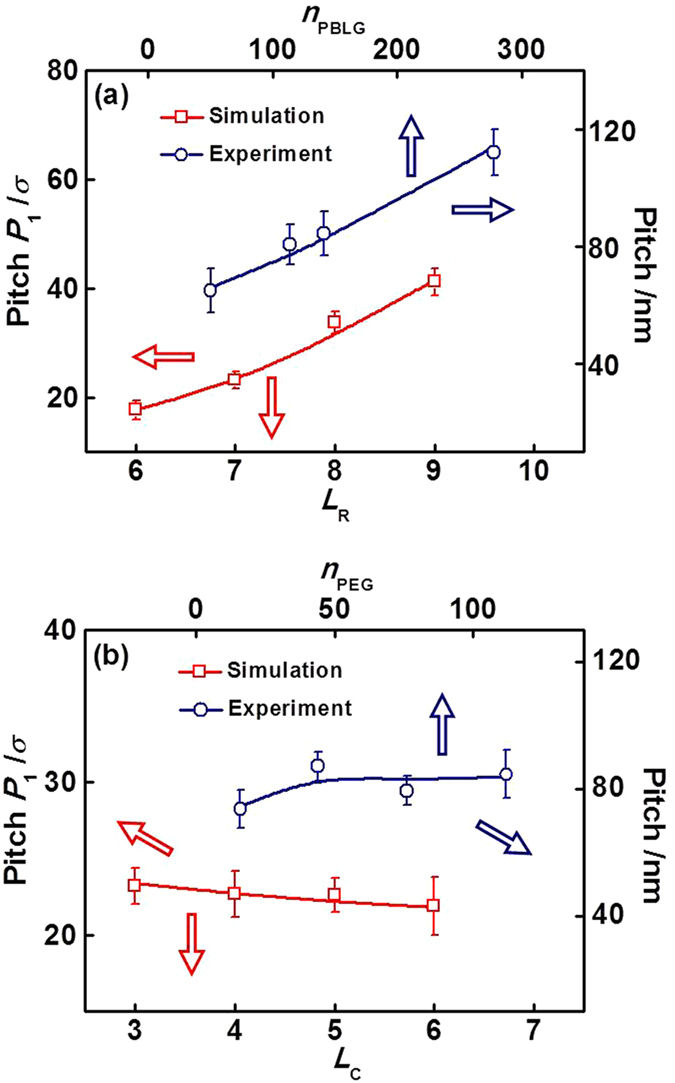
Simulated screw pitches *P*_1_ of helical structures formed by **R**_*m*_**C**_*n*_/**R**_150_ mixtures at *ε*_RR_ = 2.2 and experimentally measured pitches of super-helices from PBLG-*b*-PEG/PBLG_2411_ mixtures: (**a**) at various *L*_R_ and polymerization degrees *n*_PBLG_ of PBLG in PBLG-*b*-PEG copolymer; (**b**) at various *L*_C_ and polymerization degrees *n*_PEG_ of PEG. In figure (**a**), *L*_C_ = 3 and *n*_PEG_ = 112, while in figure (b), *L*_R_ = 7 and *n*_PBLG_ ≈ 150.

The simulation and experiment results regarding the screw pitches of helices were also compared. The simulation results shown in Fig. 5 are replotted in Fig. 8 for a comparison with experiments. As shown in Fig. 8a, the simulated pitch *P*_1_ of helical structures at *ε*_RR_ = 2.2 increases gradually as the rod block length *L*_R_ becomes larger. In the experiments, we measured the screw pitches of super-helices self-assembled from PBLG-*b*-PEG_112_/PBLG_2411_ mixtures with various polymerization degrees of PBLG (*n*_PBLG_) in PBLG-*b*-PEG copolymer by collecting a number of micelles from SEM images. The value of *n*_PBLG_ corresponds to the length of rod blocks (the experimental details are available in section 3 of Supporting Information). It was found that the experimental screw pitch increases with increasing *n*_PBLG_, which is in qualitative accordance with the simulation calculations. Figure 8b shows the simulated *P*_1_ as a function of *L*_C_, and experimental screw pitches with respect to the polymerization degree of PEG (*n*_PEG_). The *P*_1_ has a slight decrease as *L*_C_ increases, and the experimental screw pitch is also slightly affected by *n*_PEG_ (for details, see section 3 of Supporting Information). The tiny difference between them may be due to the statistical deviation. Our simulations can not only reproduce the general features of self-assembly of PBLG-*b*-PEG/PBLG mixtures, but also provide chain packing information and allow us to get a deep insight into the formation mechanism of these hierarchical assemblies.

## Discussion

Based on the simulation results, the physical principle of structural transition governed by interaction strength *ε*_RR_ was proposed. A transition from plain fibers to helical structures to abacus-like structures was found with increasing *ε*_RR_. The interesting structures are the helical structures, which are formed at moderate values of *ε*_RR_. The reason that the helical structures appear instead of abacus-like structures (higher *ε*_RR_) and plain fibers (lower *ε*_RR_) at moderate *ε*_RR_ can be explained as follows. Provided that the abacus-like structures are formed, the translational entropy of block copolymers is lost as compared with the helical structures. This is because the rod blocks tend to be packed more ordered in the abacus-like structures than that in helical structures. Although the interfacial enthalpy is more favorable, it still cannot compensate for the loss of translational entropy, and thus the total free energy increases. On the other hand, if the plain fibers are formed, the rod blocks can be distributed more dispersedly than that in helical structures, and thus the translational entropy is more favorable. However, the interfacial enthalpy dramatically increases due to the dispersed packing of rod blocks, leading to the increase of total free energy. Therefore, a balance between the translational entropy and interfacial enthalpy decides the helical structures at moderate values of *ε*_RR_. The formation of other structures at other *ε*_RR_ is also due to the similar mechanism.

It should be noted that in the simulations, the chirality of polymers is not considered and the system is treated as a blend of achiral block copolymers and achiral homopolymers, although the polymers are chiral in the experiments. Due to the achiral feature of the simulation models, the co-existence of left-handed and right-handed helical structures could exist in the simulations, and in principle there should be equal number of left-handed and right-handed helical structures in a given system.

Regarding the effect of block length on the aggregate structures, the changes of rod and coil block length can bring about changes of balance between the interfacial enthalpy and entropy loss. As the rod block length *L*_R_ increases, the hydrophobic blocks are more exposed to the solvents, inducing the interfacial enthalpy to increase. To maintain the system balance, the screw pitch *P*_1_ of helical structures and twisting pitch *P*_2_ of rod blocks both increase with increasing *L*_R_ as shown in Fig. 5a (decreasing the entropy loss). On the other hand, with increasing the coil block length *L*_C_, the coil blocks can cover the hydrophobic blocks more effectively, and the interfacial enthalpy decreases. However, the higher density of coil blocks at the rod-coil interface makes the entropy loss increase. Under the interplay between interfacial enthalpy and entropy loss, the pitches *P*_1_ and *P*_2_ have a slight change with increasing *L*_C_ (see Fig. 5b).

Cooperative self-assembly of multi-component polymers provides a promising strategy to prepare complex aggregates with controlled structures. The multi-component polymers exhibit prominent advantages over single-component polymers in achieving controllable and multi-functional properties, facilitating the applications such as in drug delivery and nanoreactors. In this work, we reported the cooperative self-assembly of rod-coil block copolymers and rigid homopolymers. A variety of hierarchical nanostructures were predicted, such as helical and abacus-like structures. The helices with a homopolymer bundles covered by block copolymer chains are reminiscent of the structure of tobacco mosaic virus, in which proteins spontaneously assemble around the RNA template. The present research on the formation mechanism of hierarchical assemblies can be of practical significance for the construction of complicated biological analogs such as a model virus and subsequent investigation of its physiological behavior, *e.g.*, cell penetration. Understanding the cooperative self-assembly of multi-component polymers also provides important information for the design and fabrication of advanced materials.

*In summary*, we applied Brownian dynamics simulations to investigate the self-assembly behavior of mixture systems containing rod-coil block copolymers and rigid homopolymers. The aggregate morphologies of mixture systems were found to be dependent on the interaction parameter *ε*_RR_, rod block length *L*_R_, coil block length *L*_C_, and mixture ratio *φ*. At higher *ε*_RR_, an abacus-like structure is formed. With decreasing *ε*_RR_, the morphology is transformed to helix, plain fiber, and then to unimers. The ordering information of rod blocks was provided. For the super-helices, two levels of the chain ordering were discovered. The block copolymers were helically wrapped on the homopolymer bundles to form helical string. Meanwhile the packing mode of the rod block exhibited a twisting manner. Based on simulation results, we constructed a phase diagram of morphology stability regions in space of *L*_R_ and *ε*_RR_ (or *L*_C_ and *ε*_RR_). The screw pitches of the helical structures and twisting pitches of rod blocks were also calculated. The pitches increase with increasing *L*_R_, while they decrease slightly as *L*_C_ increases. In addition, the mixture ratio *φ* also affects the pitches of helices. The simulation results were compared with experimental observations, and an agreement was obtained. The present work provides an insight into the formation mechanism of complex structures self-assembled from rod-coil block copolymer/rigid homopolymer mixtures.

## Methods

The simulations were carried out by applying the simulator, coarse-grained molecular dynamics program (COGNAC) of OCTA. The simulator was developed by Doi’s group, which is public on a Web site^48^. The reduced units used in COGNAC can be converted to real ones, which have been described in our previous work^41^.

We considered a mixture system containing rod-coil diblock copolymers and rigid homopolymers, and constructed a coarse-grained model. The rod-coil block copolymer was modeled as a linear chain with *m* **R** beads of rod block and *n* **C** beads of coil block, denoted by the type of **R**_*m*_**C**_*n*_. The rigid homopolymer molecule was represented by a rigid chain with *x* **R** beads (denoted by **R**_*x*_). Figure 1a shows the illustration of **R**_*m*_**C**_*n*_ block copolymer/**R**_*x*_ homopolymer mixture (for instance, **R**_7_**C**_3_/**R**_12_ mixture) model. The beads colored by red, green, and blue are hydrophobic rod block, hydrophilic coil block, and hydrophobic homopolymer, respectively.

To construct rod-coil block copolymer and rigid homopolymer molecules, potentials that should be given are bonding potential *U*_mol_ and nonbonding potential *U*_*ij*_. The former can construct a desired molecule from atoms, while the latter describes intermolecular interactions. For rigid segments, the *U*_mol_ is composed of bond stretching potential *U*_bond_(*r*) and angle bending potential *U*_angle_(*θ*). For flexible segments, there is no angle bending potential.

The bond stretching potential is a function of distance *r* between the chemically bonded beads, which is given by a harmonic potential


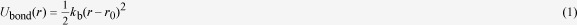


where *k*_b_ is the bond spring constant and *r*_0_ is the equilibrium bond length. In this work, the value of *k*_b_ was set to be 10000 for all bonds to avoid the over-stretching of bonds. The bond length *r*_0_ was set as 0.75 and 1.0 for the rod and coil blocks. The angle bending potential *U*_angle_(*θ*) for the rod block is given by a cosine harmonic function of the angle *θ* between every two consecutive bonds:


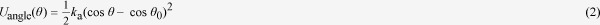


where *k*_a_ is the angle spring constant, and *θ*_0_ is the equilibrium angle. The larger the *k*_a_ value, the more rigidity of the molecule chain. The magnitude of the constant *k*_a_ was set to be 10000 in all cases. To realize the rod block, the equilibrium angle *θ*_0_ was set as a value of 0.1° (close to zero), which is not zero so that the bond angle can be adjusted. More detailed information of the choice of simulation parameters can be found in our previous work^41^.

The nonbonding potential *U*_*ij*_ is given by the standard Lennard-Jones 12:6 potential acting between any pair of beads *i* and *j*:


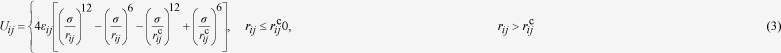


where 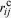 is the cutoff distance, 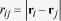 with **r**_*i*_ and **r**_*j*_ being the position vectors of the *i*-th and *j*-th beads, and *ε*_*ij*_ is the interaction parameter between beads *i* and *j*. The amphiphilicity of polymer blocks in this model is realized by introducing different cutoff distances of LJ potential^49^,^50^. The distances 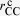, 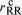, and 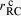
were respectively set as 2^1/6^, 2.5, and 2^1/6^, indicating that the **C**-**C** and **R**-**C** interactions are repulsive, while the **R**-**R** interaction is attractive. The selection of 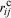 can make the **R** blocks hydrophobic and **C** blocks hydrophilic. The diameter *σ* of LJ bead is kept at unity for any pair of species. The interaction *ε*_RR_ between the **R** blocks is variable, while the other interactions are unity (*i.e.*, *ε*_RC_ = *ε*_CC_ = 1.0).

All the simulations were performed on a cubic cell (150 × 150 × 150) using a Brownian dynamics algorithm with the temperature controlling method (NVT ensemble)^51^. The beads are coupled to a heat bath, and the equations of motion are written by


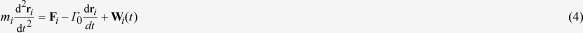


where *m*_*i*_ is the mass of the *i*-th bead, Γ_0_ is the friction constant, and **F**_*i*_ is the force acting on the *i*-th bead calculated by the potential energies consisting of *U*_mol_ and *U*_*ij*_. In the Brownian dynamics, the effect of solvent molecules is implicitly treated by a noise term **W**_*i*_(*t*), which can be calculated through the fluctuation-dissipation relation^52^:


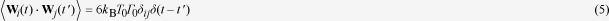


Periodic boundary conditions were imposed to minimize the effect of finite system size. A simple cubic packing mode for **R** beads and a body-centered-cubic (bcc) packing mode for **C** beads were applied to generate the initial structures of molecules, where **R**_7_**C**_3_ copolymers are located regularly around **R**_150_ homopolymer chains, and the structure relaxation was done by stochastic dynamic simulation^41^. Different initial states of the simulation system have been examined to have negligible influence on the calculation results under the present conditions employed. In the calculation, the integration time step ∆*t* = 0.004 was selected. The lengths of simulation runs were 5 × 10^6^ time steps (*i.e.*, 20000 time units), which ensured that the simulated system reached equilibrium. All calculations were performed at a temperature *T* = 3.0.

In addition, the orientation degree of rod blocks in the aggregates can be characterized by order parameter *S*. The *S*_*i*_ for *i*-th rod block is defined by:


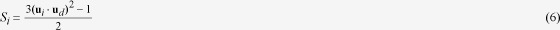


where **u**_*i*_ is the normalized vector of *i*-th rod block (from its first bead to the last bead), and **u**_*d*_ is the normalized vector of orientation direction, which is determined by iteration to find the maximum value of order parameter *S*. The order parameter *S* of rod blocks within the core is the average value of *S*_*i*_. We also present a mathematical method to describe the local twisting packing of rod blocks in the helical string. The rod alignment is characterized by the vector product **u**_*i*_**·u**_*d*_, which reflects the angle *θ*_*i*_ between the *i-*th rod and orientation direction of rod blocks, expressed as


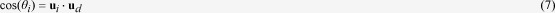


The twisting of rod blocks can be described by cos(*θ*_*i*_) as a function of position of the rod block along the major axis of the helical string. The position of rod block is defined as the projection of the mass center of each rod block onto the major axis of helical string. For a rigid fiber, the major axis can be illustrated by the homopolymer bundle axis, while for a slightly flexible fiber, the major axis should be divided into 2 or 3 subaxes^41^. The twisting pitch can be calculated from the plot of cos(*θ*) *versus* position along the axis.

## Author Contributions

Y. L. and T. J. carried out the simulations and wrote the manuscript. C. C. and X. Z. designed and carried out the experiments. S. L. and J. L. directed the research and revised the manuscript. All authors reviewed the manuscript.

## Additional Information

**How to cite this article**: Li, Y. *et al.* Hierarchical Nanostructures Self-Assembled from a Mixture System Containing Rod-Coil Block Copolymers and Rigid Homopolymers. *Sci. Rep.* **5**, 10137; doi: 10.1038/srep10137 (2015).

## Supplementary Material

Supplementary Information

## References

[b1] Wang J., Guo K., An L., Müller M. & Wang Z. Micelles of coil-comb block copolymers in selective solvents: competition of length scales. Macromolecules 43, 2037–2041 (2010).

[b2] Lorenzo A. T., Müller A. J., Priftis. D., Pitsikalis M. & Hadjichristidis N. Synthesis and morphological characterization of miktoarm star copolymers (PCL)_2_(PS)_2_ of poly *(ε*-caprolactone) and polystyrene. J. Polym. Sci., Part A: Polym. Chem. 45, 5387–5397 (2007).

[b3] Blanazs A., Armes S. P. & Ryan A. J. Self-assembled block copolymer aggregates: from micelles to vesicles and their biological applications. Macromol. Rapid. Commun. 30, 267–277 (2009). 21706604 10.1002/marc.200800713

[b4] Pispas S. & Sarantopoulou E. Self-assembly in mixed aqueous solutions of amphiphilic block copolymers and vesicle-forming surfactant. Langmuir 23, 7484–7490 (2007). 17559242 10.1021/la700342s

[b5] Liu J. *et al.* The *in vitro* biocompatibility of self-assembled hyperbranched copolyphosphate nanocarriers. Biomaterials 31, 5643–5651 (2010). 20417961 10.1016/j.biomaterials.2010.03.068

[b6] van Dongen S. *et al.* Biohybrid polymer capsules. Chem. Rev. 109, 6212–6274 (2009). 19663429 10.1021/cr900072y

[b7] Jenekhe S. A. & Chen X. L. Self-assembled aggregates of rod-coil block copolymers and their solubilization and encapsulation of fullerenes. Science 279, 1903–1907 (1998). 9506934 10.1126/science.279.5358.1903

[b8] Klok H.-A. & Lecommandoux S. Supramolecular materials *via* block copolymer self-assembly. Adv. Mater. 13, 1217–1229 (2001).

[b9] Harada A. & Kataoka K. Formation of polyion complex micelles in an aqueous milieu from a pair of oppositely-charged block copolymers with poly(ethylene glycol) segments. Macromolecules 28, 5294–5299 (1995).

[b10] Zhu J. & Hayward R. C. Wormlike micelles with microphase-separated cores from blends of amphiphilic AB and hydrophobic BC diblock copolymers. Macromolecules 41, 7794–7797 (2008).

[b11] Li Z., Hillmyer M. A. & Lodge T. P. Control of structure in multicompartment micelles by blending *μ*-ABC star terpolymers with AB diblock copolymers. Macromolecules 39, 765–771 (2006).

[b12] Luo L. & Eisenberg A. One-step preparation of block copolymer vesicles with preferentially segregated acidic and basic corona chains. Angew. Chem., Int. Ed. 41, 1001–1004 (2002). 10.1002/1521-3773(20020315)41:6<1001::aid-anie1001>3.0.co;2-q 12491293

[b13] Hu J. & Liu G. Chain mixing and segregation in B-C and C-D diblock copolymer micelles. Macromolecules 38, 8058–8065 (2005).

[b14] Li G., Shi L., Ma R., An Y. & Huang N. Formation of complex micelles with double-responsive channels from self-assembly of two diblock copolymers. Angew. Chem., Int. Ed. 45, 4959–4962 (2006). 10.1002/anie.200600172 16807950

[b15] Hui T., Chen D. & Jiang M. A one-step approach to the highly efficient preparation of core-stabilized polymeric micelles with a mixed shell formed by two incompatible polymers. Macromolecules 38, 5834–5837 (2005).

[b16] Yan X., Liu G., Hu J. & Willson C. G. Coaggregation of B-C and D-C diblock copolymers with H-bonding C blocks in block-selective solvents. Macromolecules 39, 1906–1912 (2006).

[b17] Štěpánek M. *et al.* Hybrid polymeric micelles with hydrophobic cores and mixed polyelectrolyte/nonelectrolyte shells in aqueous media. 1. preparation and basic characterization. Langmuir 17, 4240–4244 (2001).

[b18] Davis K. P., Lodge T. P. & Bates F. S. Vesicle membrane thickness in aqueous dispersions of block copolymer blends. Macromolecules 41, 8289–8291 (2008).

[b19] Mingvanish W., Chaibundit C. & Booth C. Mixed micellisation of oxyethylene-oxybutylene diblock and triblock copolymers in water studied by light scattering. Phys. Chem. Chem. Phys. 4, 778–784 (2002).

[b20] Koňák C. & Helmstedt M. Comicellization of diblock and triblock copolymers in selective solvents. Macromolecules 36, 4603–4608 (2003).

[b21] Gohy J.-F., Varshney S. K. & Jérôme R. Water-soluble complexes formed by poly(2-vinylpyridinium)-*block*-poly(ethylene oxide) and poly(sodium methacrylate)-*block*-poly(ethylene oxide) copolymers. Macromolecules 34, 3361–3366 (2001).

[b22] Zhuang Z., Zhu X., Cai C., Lin J. & Wang L. Self-assembly of a mixture system containing polypeptide graft and block copolymers: experimental studies and self-consistent field theory simulations. J. Phys. Chem. B 116, 10125–10134 (2012). 22838739 10.1021/jp305956v

[b23] Luo K. *et al.* Biodegradable interpolyelectrolyte complexes based on methoxy poly(ethylene glycol)-*b*-poly(*α*,L-glutamic acid) and chitosan. Biomacromolecules 9, 2653–2661 (2008). 18754685 10.1021/bm800767f

[b24] Cai C., Lin J., Chen T., Wang X. & Lin S. Super-helices self-assembled from a binary system of amphiphilic polypeptide block copolymers and polypeptide homopolymers. Chem. Commun. 2709–2711, 10.1039/b823367e (2009). 19532929

[b25] Cai C. *et al.* Simulation-assisted self-assembly of multicomponent polymers into hierarchical assemblies with varied morphologies. Angew. Chem., Int. Ed. 52, 7732–7736 (2013). 10.1002/anie.201210024 23775798

[b26] Lin J. *et al.* Drug releasing behavior of hybrid micelles containing polypeptide triblock copolymer. Biomaterials 30, 108–117 (2009). 18838162 10.1016/j.biomaterials.2008.09.010

[b27] Tang D. *et al.* Self-assembly of poly*(γ*-benzyl-L-glutamate)-*graft*-poly(ethylene glycol) and its mixtures with poly*(γ*-benzyl-L-glutamate) homopolymer. Macromol. Rapid Commun. 25, 1241–1246 (2004).

[b28] Li T., Lin J., Chen T. & Zhang S. Polymeric micelles formed by polypeptide graft copolymer and its mixtures with polypeptide block copolymer. Polymer 47, 4485–4489 (2006).

[b29] Chen L., Jiang T., Lin J. & Cai C. Toroid formation through self-assembly of graft copolymer and homopolymer mixtures: experimental studies and dissipative particle dynamics simulations. Langmuir 29, 8417–8426 (2013). 23738828 10.1021/la401553a

[b30] Chécot F., Lecommandoux S., Gnanou Y. & Klok H.-A. Angew. Chem., Int. Ed. 41, 1339–1343 (2002). 10.1002/1521-3773(20020415)41:8<1339::aid-anie1339>3.0.co;2-n 19750757

[b31] Kukula H., Schlaad H., Antonietti M. & Förster S. The formation of polymer vesicles or “Peptosomes” by polybutadiene-*block*-poly(L-glutamate)s in dilute aqueous solution. J. Am. Chem. Soc. 124, 1658–1663 (2002). 11853440 10.1021/ja012091l

[b32] Cai C., Lin J., Chen T. & Tian X. Aggregation behavior of graft copolymer with rigid backbone. Langmuir 26, 2791–2797 (2010). 20141213 10.1021/la902834m

[b33] Ma J., Li X., Tang P. & Yang Y. Self-assembly of amphiphilic ABC star triblock copolymers and their blends with AB diblock copolymers in solution: self-consistent field theory simulations. J. Phys. Chem. B 111, 1552–1558 (2007). 17266363 10.1021/jp067650v

[b34] Zhuang Y., Lin J., Wang L. & Zhang L. Self-assembly behavior of AB/AC diblock copolymer mixture in dilute solution. J. Phys. Chem. B 113, 1906–1913 (2009). 19170547 10.1021/jp809181d

[b35] Xu G., Feng X. & Li Y. Self-assembled nanostructures of homopolymer and diblock copolymer blends in a selective solvent. J. Phys. Chem. B 114, 1257–1263 (2010). 20050622 10.1021/jp908823h

[b36] Li X., Tang P., Qiu F., Zhang H. & Yang Y. Aggregates in solution of binary mixtures of amphiphilic diblock copolymers with different chain length. J. Phys. Chem. B 110, 2024–2030 (2006). 16471778 10.1021/jp055951j

[b37] Srinivas G. & Pitera J. W. Soft patchy nanoparticles from solution-phase self-assembly of binary diblock copolymers. Nano Lett. 8, 611–618 (2008). 18189443 10.1021/nl073027q

[b38] Xin J., Liu D. & Zhong C. Morphology and structure control of multicompartment micelles from triblock copolymer blends. J. Phys. Chem. B 113, 9364–9372 (2009). 19537743 10.1021/jp902300g

[b39] Sens P., Marques C. M. & Joanny J.-F. Mixed micelles in a bidisperse solution of diblock copolymers. Macromolecules 29, 4880–4890 (1996).

[b40] Palyulin V. V. & Potemkin I. I. Mixed versus ordinary micelles in the dilute solution of AB and BC diblock copolymers. Macromolecules 41, 4459–4463 (2008).

[b41] Lin S., Numasawa N., Nose T. & Lin J. Brownian molecular dynamics simulation on self-assembly behavior of rod-coil diblock copolymers. Macromolecules 40, 1684–1692 (2007).

[b42] Lin S., He X., Li Y., Lin J. & Nose T. Brownian molecular dynamics simulation on self-assembly behavior of diblock copolymers: influence of chain conformation. J. Phys. Chem. B 113, 13926–13934 (2009). 19788196 10.1021/jp904707a

[b43] Li Y., Lin S., He X., Lin J. & Jiang T. Self-assembly behavior of ABA coil-rod-coil triblock copolymers: a Brownian dynamics simulation approach. J. Chem. Phys. 135, 014102 (2011). 21744883 10.1063/1.3606396

[b44] Bhargava P. *et al.* Temperature-induced reversible morphological changes of polystyrene-*block*-poly(ethylene oxide) micelles in solution. J. Am. Chem. Soc. 129, 1113–1121 (2007). 17263392 10.1021/ja0653019

[b45] Ortiz V. *et al.* Dissipative particle dynamics simulations of polymersomes. J. Phys. Chem. B 109, 17708 (2005). 16853266 10.1021/jp0512762

[b46] Ding W., Lin S., Lin J. & Zhang L. Effect of chain conformational change on micelle structures: experimental studies and molecular dynamics simulations. J. Phys. Chem. B 112, 776 (2008). 18154327 10.1021/jp076939p

[b47] Jeppesen C. *et al.* Impact of polymer tether length on multiple ligand-receptor bond formation. Science 293, 465 (2001). 11463908 10.1126/science.293.5529.465

[b48] OCTA Home Page. http://octa.jp (Accessed: 8th January 2007).

[b49] Iacovella C. R. & Glotzer S. C. Complex crystal structures formed by the self-assembly of ditethered nanospheres. Nano Letters 9, 1206–1211 (2009). 19215081 10.1021/nl900051u

[b50] Phillips C. L., Iacovella C. R. & Glotzer S. C. Stability of the double gyroid phase to nanoparticle polydispersity in polymer-tethered nanosphere systems. Soft Matter 6, 1693–1703 (2010).

[b51] Grest G. S. & Kremer K. Molecular dynamics simulation for polymers in the presence of a heat bath. Phys. Rev. A 33, 3628–3631 (1986). 9897103 10.1103/physreva.33.3628

[b52] Grest G. S. & Lacasse M. D. Efficient continuum model for simulating polymer blends and copolymers. J. Chem. Phys. 105, 10583–10594 (1996).

