# Supplementary material for: Hierarchical Nanostructures Self-Assembled from a Mixture System Containing Rod-Coil Block Copolymers and Rigid Homopolymers
Source: Sci Rep. 2015 May 12;5:10137. doi: 10.1038/srep10137 (PMC4428031; doi:10.1038/srep10137)
Supplement: Supplementary Information [file srep10137-s1.doc]

Supporting Information

Hierarchical Nanostructures Self-Assembled from a Mixture System Containing Rod-Coil Block Copolymers and Rigid Homopolymers

*Yongliang Li, Tao Jiang,* *Shaoliang Lin,[[1]](#footnote-2)* Jiaping Lin,*^*^ *Chunhua Cai & Xingyu Zhu*

*Shanghai Key Laboratory of Advanced Polymeric Materials, State Key Laboratory of Bioreactor Engineering, Key Laboratory for Ultrafine Materials of Ministry of Education, School of Materials Science and Engineering, East China University of Science and Technology, Shanghai 200237, China*

**1. Synthesis and Characterization of Polymers**

**Reagents and materials.** α-Methoxy-ω-amino poly(ethylene glycol) (mPEG-NH_2_, *M*_n_ = 750, 2000, 3400, 5000) was purchased from Sigma-Aldrich. *N*-carboxyl-benzyl-L-glutamate anhydride (BLG-NCA) was synthesized according to literatures.^S1-S3^ Triethylamine was refluxed with sodium and distilled immediately before use. Dialysis bag (Membra-cel, 3500 molecular weight cut-off) was provided by Serva Electrophoresis GmbH. All other chemicals were obtained from Adamas-beta, and purified according to conventional methods or used as received.

**Synthesis of PBLG homopolymer.** Poly(*γ*-benzyl-L-glutamate) (PBLG) homopolymer was synthesized in anhydrous 1,4-dioxane solution using ring-opening polymerization of BLG-NCA initiated by anhydrous triethylamine.^S1-S3^ The monomer concentration is ca. 3.0 wt % and the polymerization lasted for 3 days at 15 ^o^C under a dry nitrogen atmosphere. At the end of the polymerization, the reaction mixture was poured into a large volume of anhydrous ethanol. The precipitates were collected and dried under vacuum. The resulting products were purified twice by repeated precipitation from a chloroform solution into a large volume of anhydrous methanol. The molecular weight of PBLG was obtained from a multi-detector gel permeation chromatography (GPC/SLS) performed at 35 ^o^C (waters 515) with DMF as mobile phase. The absolute number-average molecular weight (*M*_n_) was estimated to be 528000 by SLS and the polydispersity index (PDI) is determined to be 1.15 by GPC.

**Synthesis of PBLG-*b*-PEG block copolymers.** Poly(*γ*-benzyl-L-glutamate)-*b*-poly(ethylene glycol) (PBLG-*b*-PEG) block copolymer was synthesized by ring-opening polymerization of BLG-NCA in anhydrous 1,4-dioxane initiated by mPEG-NH_2_ macroinitiator according to our previous work.^S4,S5^ Before use, mPEG-NH2 was dried by dissolving in toluene and then removing toluene in high vacuum. The reaction was performed in flame-dried reaction bottle under a dry nitrogen atmosphere for 3 days at 15 ^o^C. The purification procedure of PBLG-*b*-PEG block copolymer is the same as that of PBLG homopolymer.

The block copolymer molecular weight was estimated using ^1^H NMR measurement (Avance 550, Bruker, CDCl_3_ as solvent). It was calculated by the peak intensities of the methylene proton signal (5.1 ppm) of PBLG and the ethylene proton signal (3.6 ppm) of PEG.^S4-S7^ For a typical sample, the *M*_n_ value of the PBLG block and PBLG-*b*-PEG block copolymer were calculated to be 31000 and 36000, respectively. The sample can be denoted by PBLG_141_-*b*-PEG_112_, where the subscripts represent the degree of polymerization for each segment. In addition to PBLG_141_-*b*-PEG_112_ sample, PBLG-*b*-PEG block copolymers with various compositions were also synthesized in the present study, such as PBLG_50_-*b*-PEG_112_, PBLG_114_-*b*-PEG_112_, PBLG_141_-*b*-PEG_112_, PBLG_278_-*b*-PEG_112_, PBLG_150_-*b*-PEG_76_, PBLG_155_-*b*-PEG_44_, and PBLG_155_-*b*-PEG_16_. The corresponding PDI values for these samples are 1.15, 1.12, 1.23, 1.21, 1.26, 1.18, and 1.19, respectively.

**Sample preparation procedure.** To prepare self-assemblies, PBLG-*b*-PEG block copolymers and PBLG homopolymers were first dissolved in tetrahydrofuran (THF)/*N*,*N*'-dimethylformamide (DMF) (3/7, v/v) mixture solvent. Usually, the polymer concentration of stock solutions is 0.3 g/L. Block copolymer and homopolymer solutions were first mixed with designed volume ratio, typically 8 mL PBLG_141_-*b*-PEG_112_ block copolymer solution and 2 mL PBLG_2411_ homopolymer solution. For the study of the effect of the mixing ratio of block copolymer to homopolymer on the self-assembled structures, the total polymer concentration is fixed at 0.3 g/L, and the feed ratio of block copolymer to homopolymer was then adjusted. To the system, deionized water (2.5 mL), a selective solvent for PEG, was added at a rate of ca. 1 mL/min with vigorous stirring. Upon the addition of water, the colorless solution gradually became tint blue, which indicates the formation of self-assembled aggregates. The solution was dialyzed against deionized water for at least 3 days to remove organic solvents.

All experimental procedures, including the processes of adding water and dialysis were performed at a constant temperature. The polymer solutions, water for micellization and water for dialysis were stored at a corresponding temperature for more than 12 h before use. The self-assembling experiments were conducted at various temperatures of 5, 20, or 40 ^o^C. It should be noted that, after the assemblies were eventually formed, the store temperature has negligible effect on the morphologies of the aggregates.

**Scanning electron microscopy (SEM) measurement.** The aggregate structures were observed by SEM. The surface profile of the aggregates was obtained from Field Emission SEM (S4800, Hitachi) operated at an accelerating voltage of 10 kV. The samples were prepared by placing drops of solution on a copper grid coated with carbon film and then were dried at room temperature. Before observation, the samples were sputtered by gold.

**2. Experiment Study on the Effect of Mixture Ratio on Aggregate Structures**

As revealed by the simulations in the main text (Figure 8), the assembled structure is markedly affected by the mixture ratio of block copolymer to homopolymers. When the molar ratio of R_7_C_3_ block copolymer to R_150_ homopolymer is higher (*φ* = 165), the helical feature is clearly visible. With decreasing the molar ratio of R_7_C_3_ to R_150_, the helical structures tend to be less visible (*φ* = 112.5), and then fibers with no helical structure but a rough surface appear (*φ* = 80). Finally, with a small molar ratio of R_7_C_3_ to R_150_ (*φ* = 52.5), the block copolymers simply covered the homopolymer bundles, and plain fibers were formed.

In addition to the simulation work, the effect of mixture ratio of PBLG-*b*-PEG block copolymer to PBLG homopolymers was further studied experimentally. The aggregate morphology of PBLG_141_-*b*-PEG_112_/PBLG_2600_ binary mixtures with molar ratios varied from 130 to 6 was studied. Figure S1 shows the scanning electron microscopy (SEM) images of the formed aggregates. The results reveal that fibers are formed in all the cases, but with different surface morphologies depending on the mixture ratio of the two polymers. For the binary systems with higher molar ratio (MR) of PBLG-*b*-PEG block copolymer to PBLG homopolymer (MR = 130), the helices is clearly visible (Figure S1a). However, as shown in Figure S1b, with decreasing the MR to 35, the depth of the screw-thread of the super-helices becomes much shallower, and the helical structures tend to be less visible. For the system with MR = 14, fibers with no helical structure but a rough surface were produced (Figure S1c). When the molar fraction of homo-PBLG is further increased, completely plain fibers are obtained as shown in Figure S1d (MR = 6).

**Figure S1** SEM images of fibers prepared from PBLG_141_-*b*-PEG_112_/PBLG_2411_ binary system with various molar ratio of PBLG-*b*-PEG block copolymer to PBLG homopolymer: (a) MR = 130; (b) MR = 35; (c) MR = 14; and (d) MR = 6. The samples were prepared at 20 ^o^C. Scale bars: 500 nm.

**3. Experiment Study on the Effect of Block Length on Aggregate Structures**

The effects of PBLG block length and PEG block length on the aggregate structures of PBLG-*b*-PEG/PBLG binary systems were examined. The degree of polymerization of PBLG homopolymers was fixed as 2411 (*M*_n_ = 528000).

(a) The effect of PBLG length. When studying the effect of PBLG block length, the degree of polymerization of PEG block was chosen to be 112 (*M*_w_ = 5000). The degrees of polymerization of PBLG blocks were changed from 50 to 278 (molecular weight *M*_n_ = 11000~61000). As shown in Figure S2, super-helices are formed from binary systems with various block copolymers consisting same PEG block length: (a) PBLG**_50_**-*b*-PEG_112_; (b) PBLG**_114_**-*b*-PEG_112_; (c) PBLG**_141_**-*b*-PEG_112_; (d) PBLG**_278_**-*b*-PEG_112_. As can be seen form the Figure, the screw pitch of the super-helices increases continuously with the molecular weight of PBLG blocks, that is from about 70 nm for PBLG**_50_**-*b*-PEG_112_/PBLG_2411_ to about 270 nm for PBLG**_278_**-*b*-PEG_112_/PBLG_2411_ helices. We then plotted the pitches of the super-helices against the polymerization degrees *n*_PBLG_ of PBLG in PBLG-*b*-PEG copolymers. The result is shown in Figure 8 of main text. The average screw pitch values of the super-helices were obtained by measuring more than 50 assemblies for each data point.

(b) The effect of PEG length (Figure S3). At a fixed PBLG block length (DP ≈ 150), super-helical structures were observed with the PEG block length varies from 16 to 112 (*M*_n_ = 750~5000). As shown in Figure S3, the length of PEG block has no significant effect on the screw pitch of the super-helices. All these samples have a screw pitch of about 80 nm. A plot of pitches against polymerization degrees *n*_PEG_ is shown in Figure 8 of main text (the data were obtained by measuring more than 50 assemblies for each point).


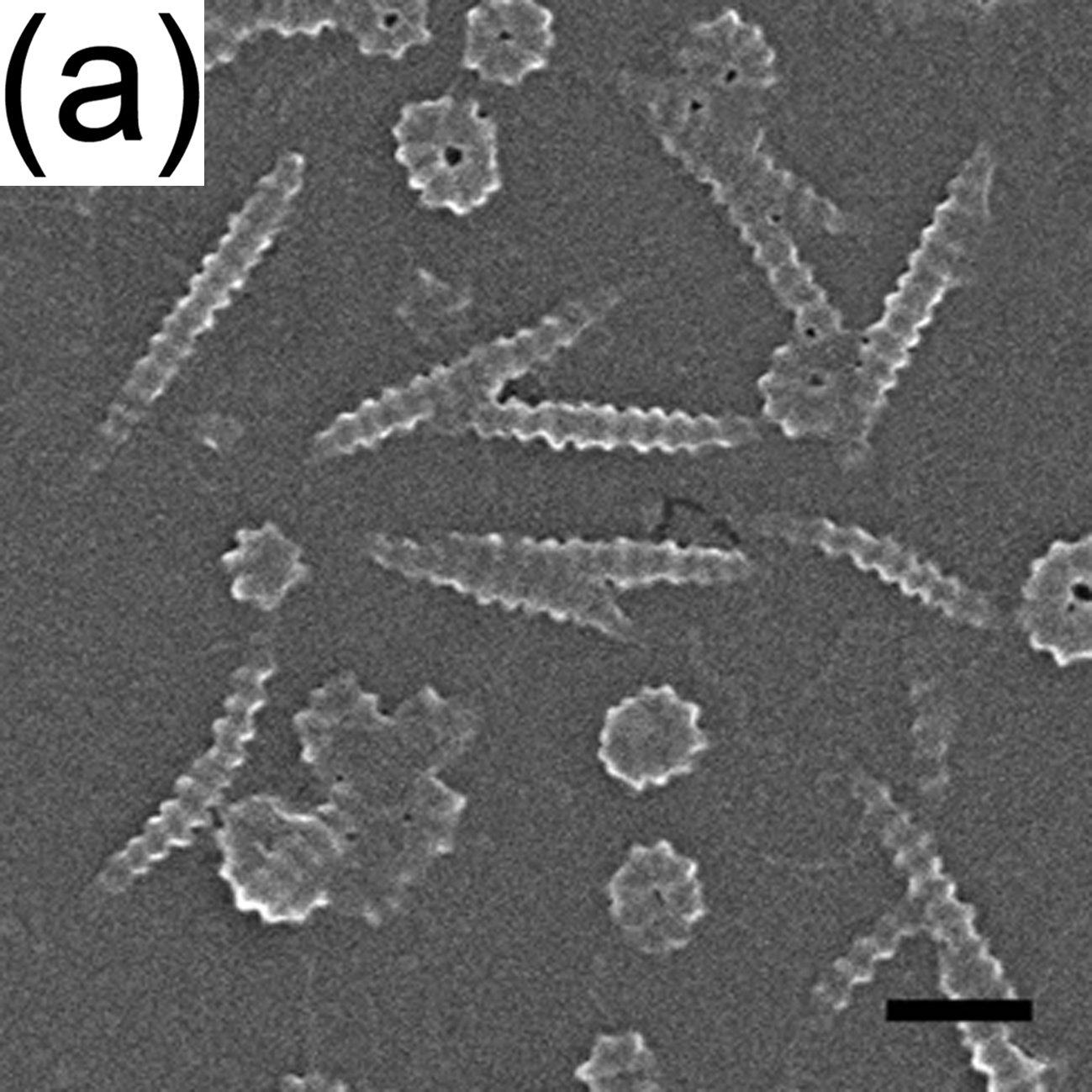

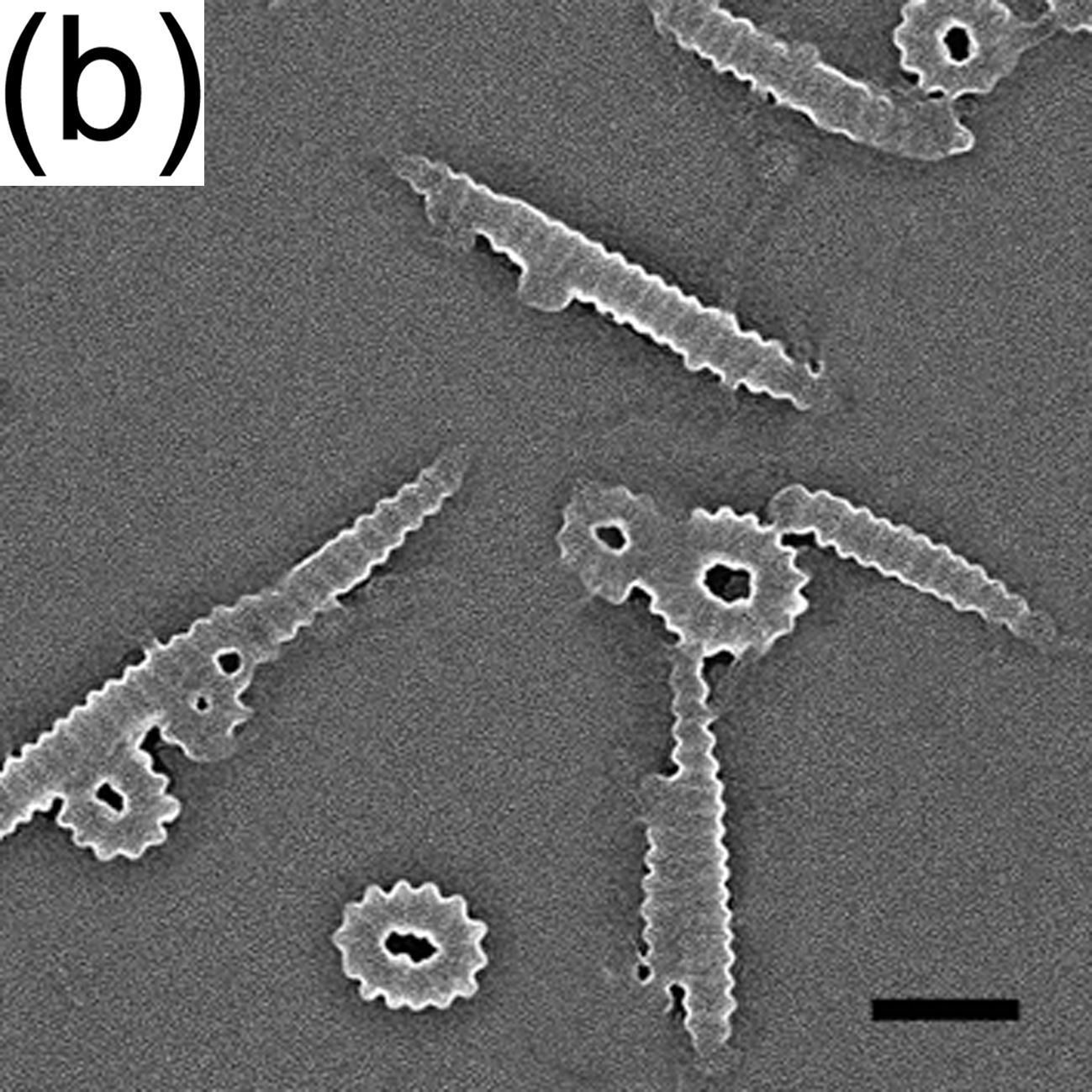


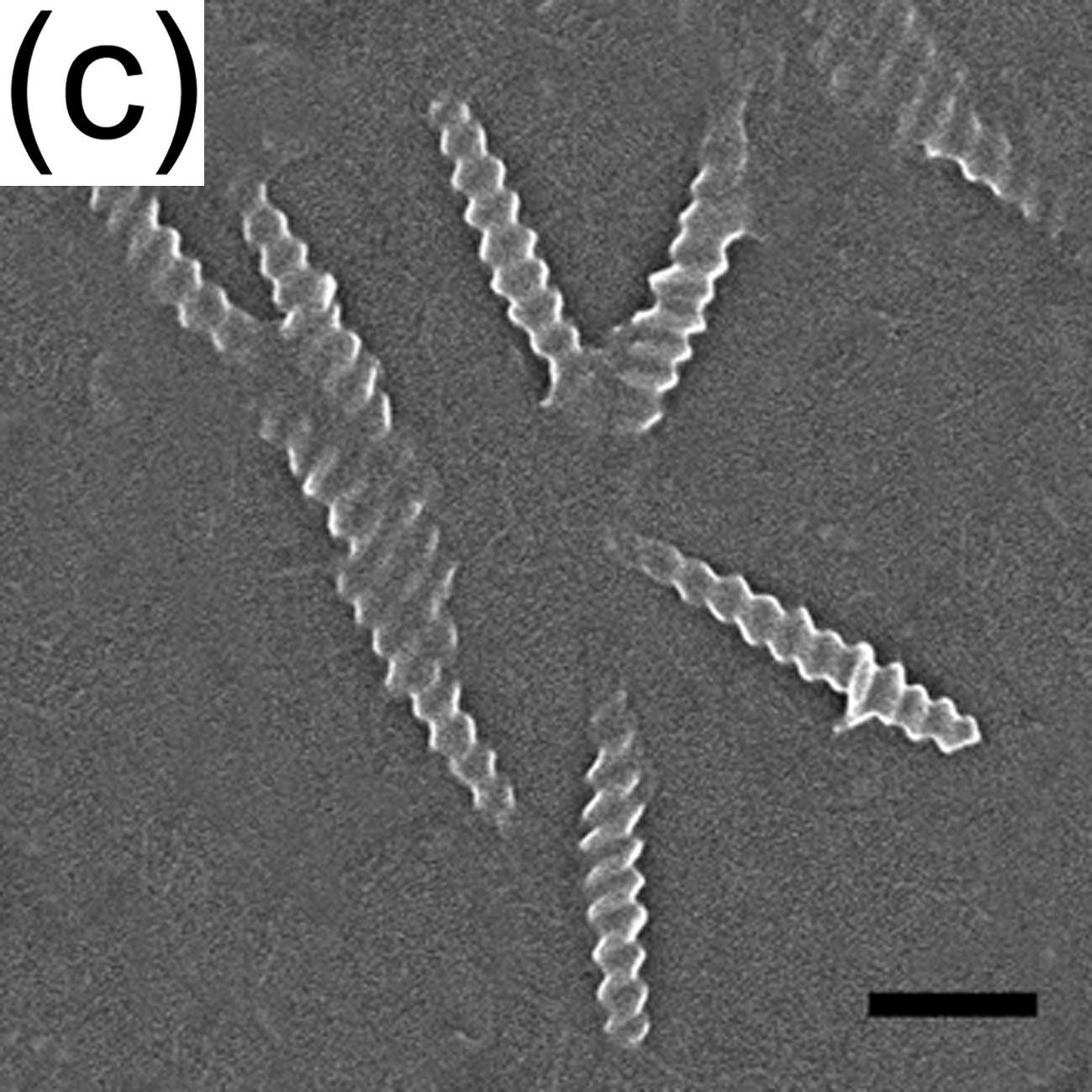

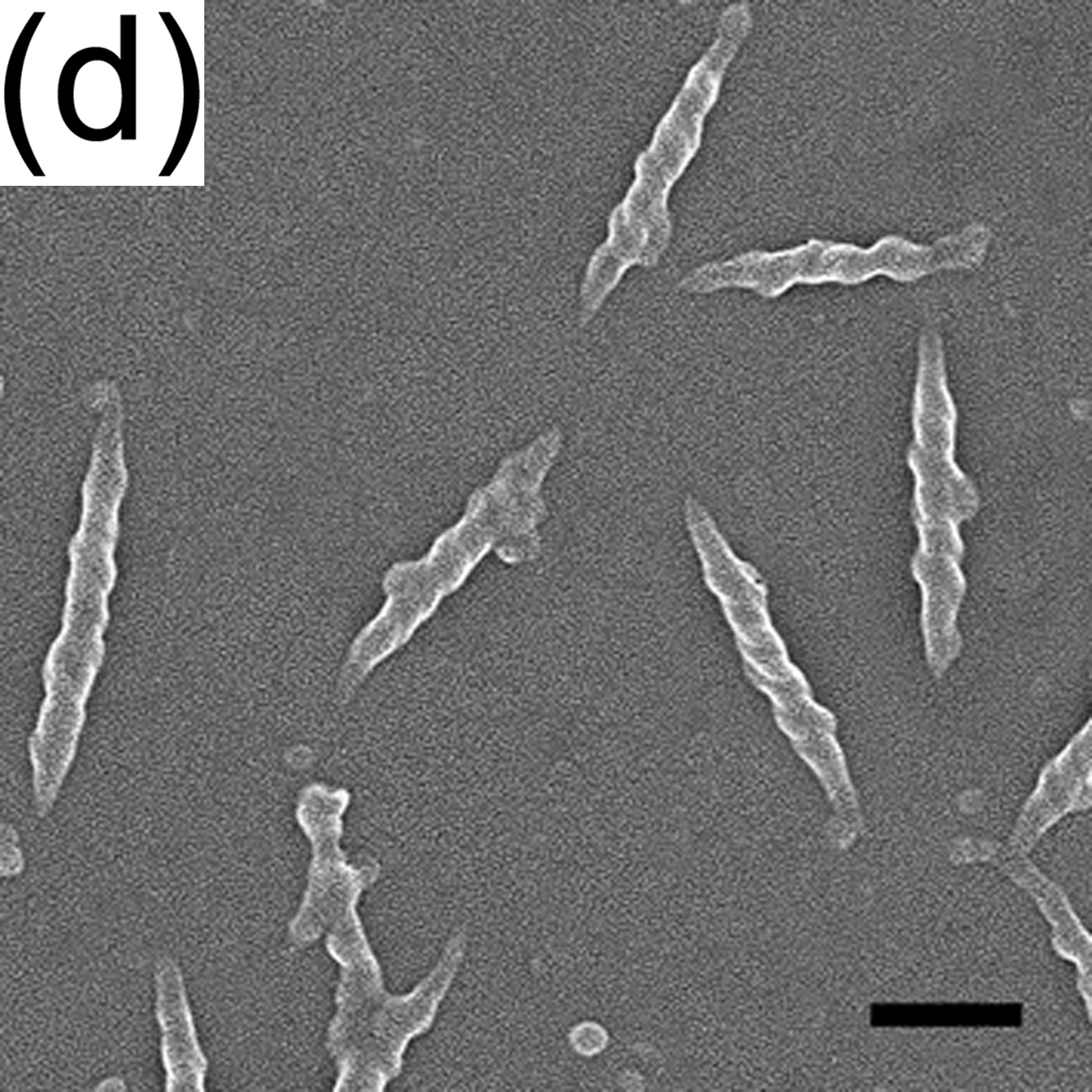


**Figure S2** The effect of PBLG block length of PBLG-*b*-PEG on the morphology of PBLG-*b*-PEG/PBLG_2411_ binary systems: (a) PBLG_50_-*b*-PEG_112_/PBLG_2411_; (b) PBLG_114_-*b*-PEG_112_/PBLG_2411_; (c) PBLG_141_-*b*-PEG_112_/PBLG_2411_; (d) PBLG_278_-*b*-PEG_112_/PBLG_2411_. The samples were prepared at 20 ^o^C. Scale bars: 400 nm.


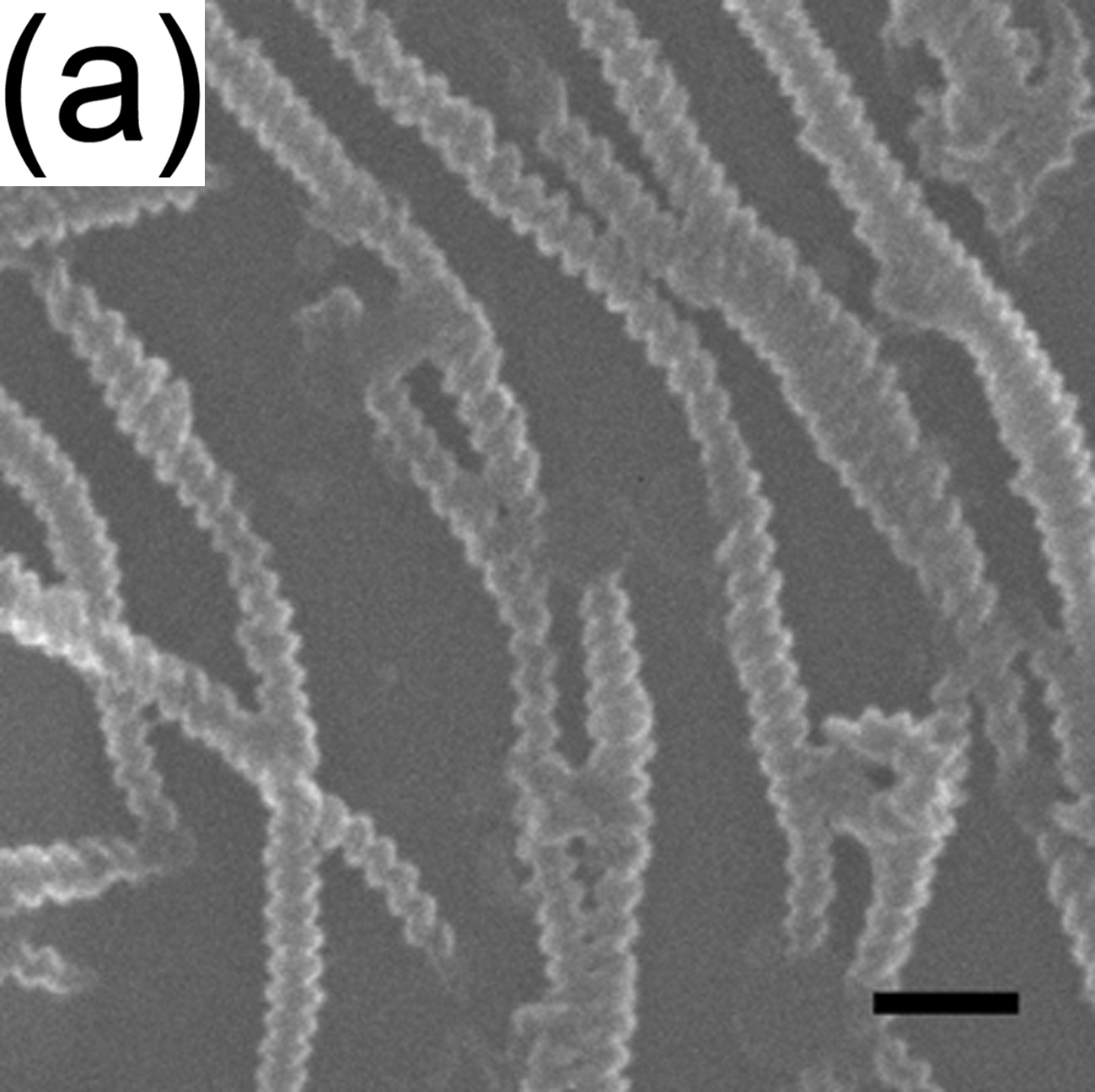

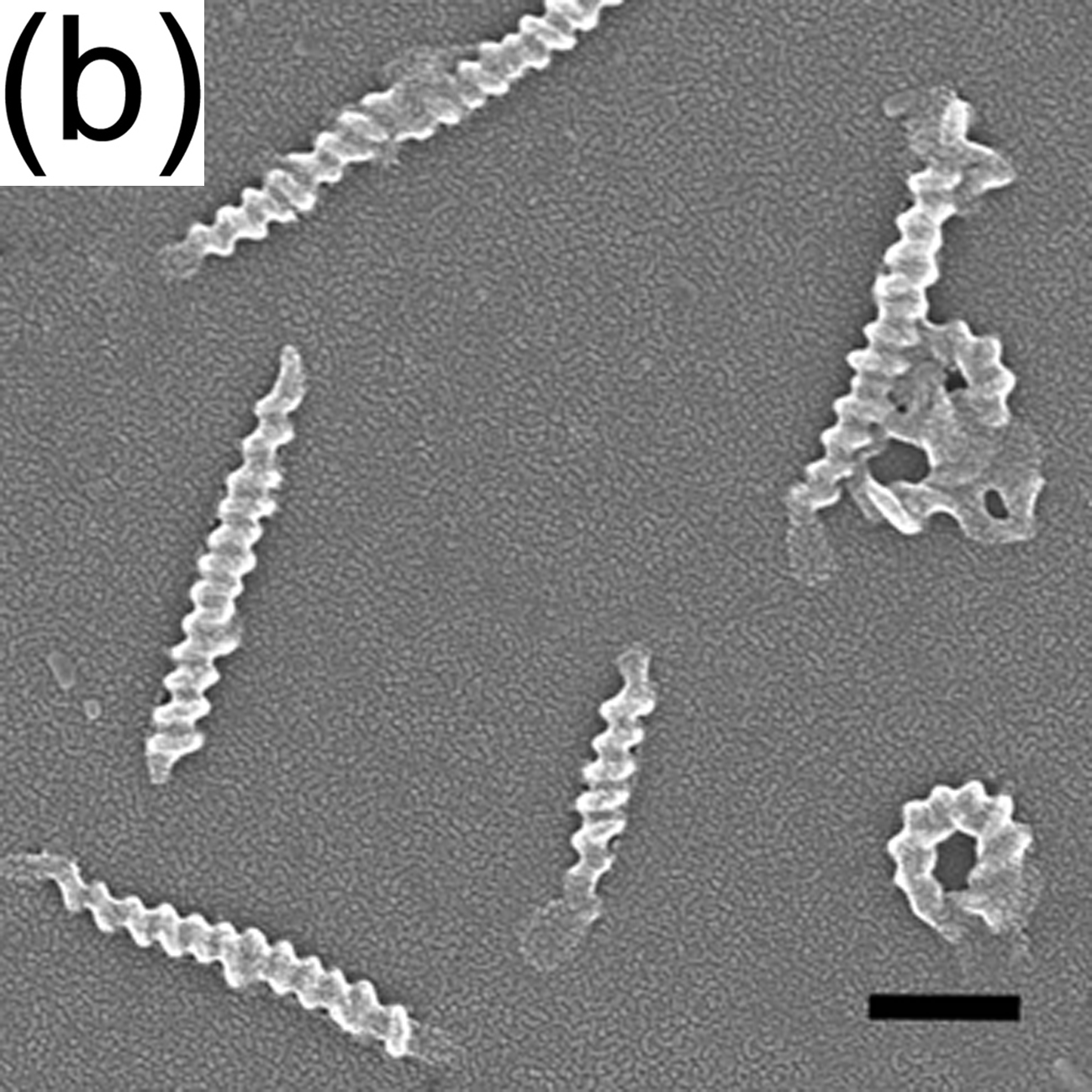


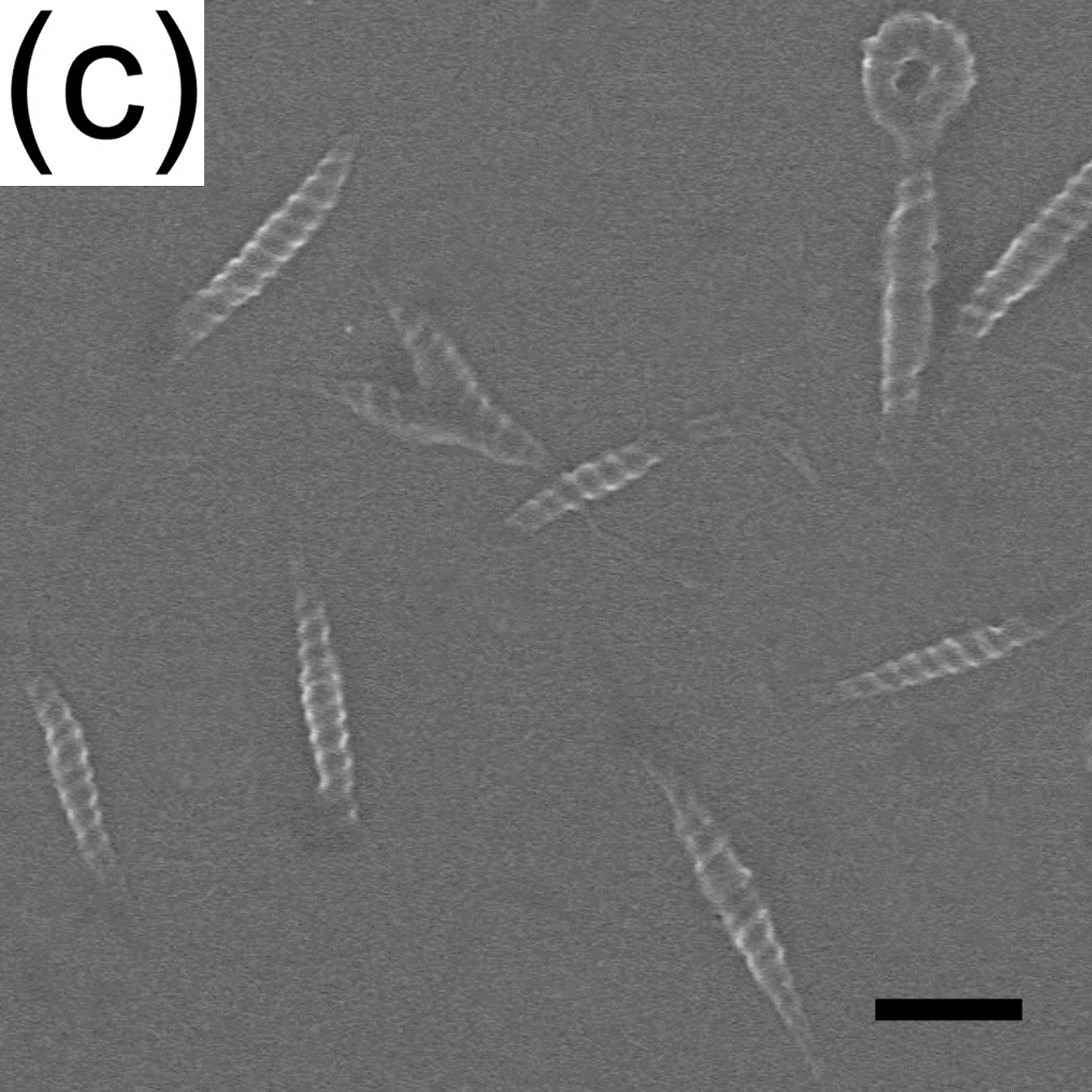

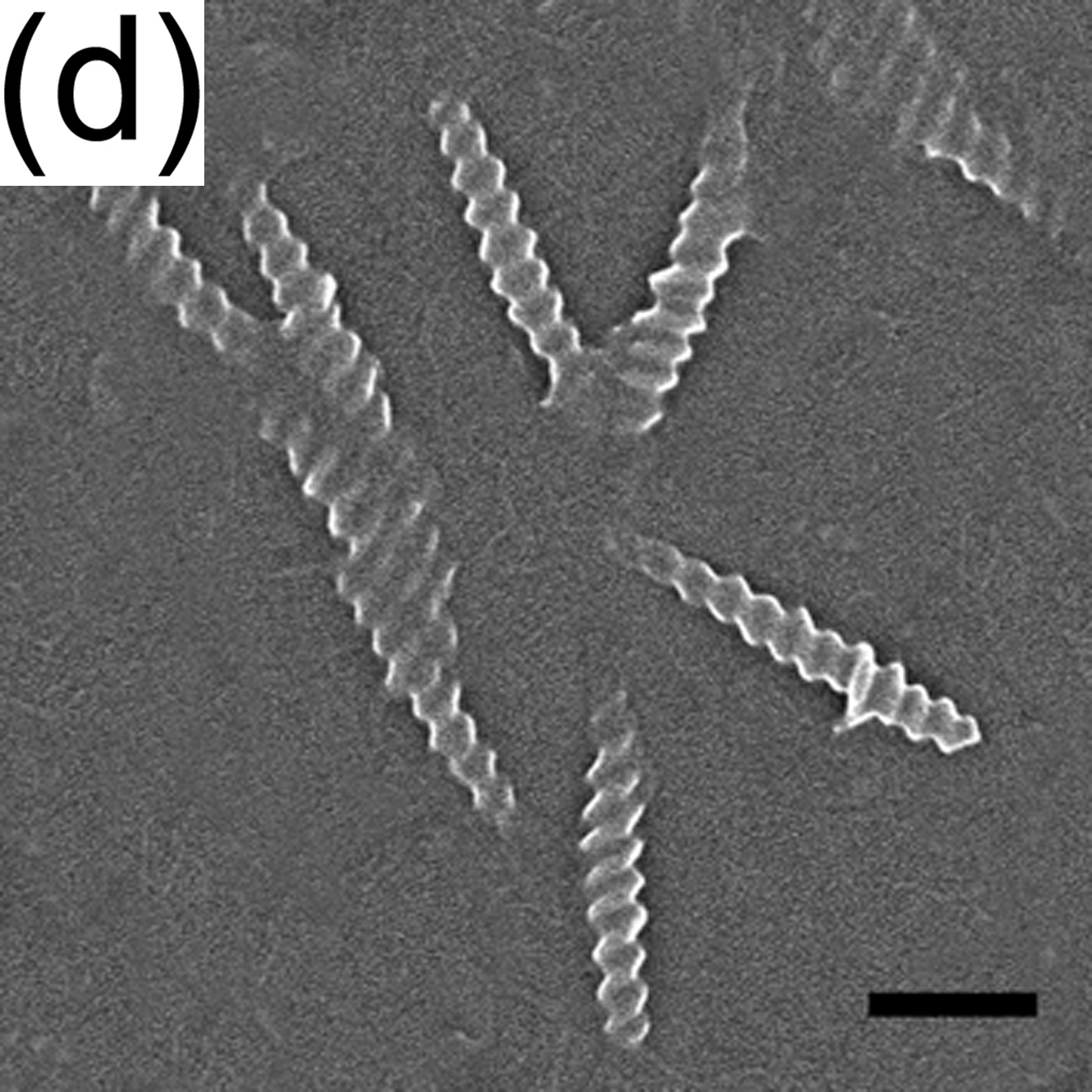


**Figure S3** The effect of PEG block length of PBLG-*b*-PEG on the morphology of PBLG-*b*-PEG/PBLG_2411_ binary systems: (a) PBLG_155_-*b*-PEG_16_/PBLG_2411_; (b) PBLG_155_-*b*-PEG_44_/PBLG_2411_; (c) PBLG_150_-*b*-PEG_76_/PBLG_2411_ (d) PBLG_141_-*b*-PEG_112_/PBLG_2411_. The samples were prepared at 20 ^o^C. Scale bars: 400 nm.

**References**

1. Lin, J., Liu, N., Chen, J. & Zhou, D. Conformational changes coupled with the isotropic-anisotropic transition: 1. experimental phenomena and theoretical considerations. *Polymer* **41**, 6189-6194 (2000).

2. Lin, J., Abe, A., Furuya, H. & Okamoto, S. Liquid crystal formation coupled with the coil-helix transition in the ternary system PBLG. *Macromolecules* **29**, 2584-2589 (1996).

3. Jeong, Y.-I., Nah, J.-W., Lee, H.-C., Kim, S.-H. & Cho, C.-S. Adriamycin release from flower-type polymeric micelle based on star-block copolymer composed of poly(*γ*-benzyl-L-glutamate) as the hydrophobic part and poly(ethylene oxide) as the hydrophilic part. *Int. J. Pharm*. **188**, 49-58 (1999).

4. Cai, C., Zhu, W., Chen, T., Lin, J. & Tian, X. Synthesis and self-assembly behavior of amphiphilic polypeptide-based brush-coil block copolymers. *J. Polym. Sci., Part A: Polym. Chem.* **47**, 5967-5978 (2009).

5. Ding, W., Lin, S., Lin, J. & Zhang, L. Effect of chain conformational change on micelle structures: experimental studies and molecular dynamics simulations. *J. Phys. Chem. B* **112**, 776-783 (2008).

6. Cho, C.-S., Nah, J.-W., Jeong, Y.-I., Cheon, J.-B., Asayama, S., Ise, H. & Akaike, T. Conformational transition of nanoparticles composed of poly(*γ*-benzyl-L-glutamate) as the core and poly(ethylene oxide) as the shell. *Polymer* **40**, 6769-6775 (1999).

7. Dimitrov, I. V., Berlinova, I. V., Iliev, P. V. & ladimirov, N. G. Controlled synthesis of peptide-based amphiphilic copolymers. *Macromolecules* **41**, 1045-1049 (2008).

1. ^*^**Corresponding Author**

   ^*^ Tel: +86-21-6425-3370.

   E-mail: jlin@ecust.edu.cn (J.L.); linshaoliang@hotmail.com (S.L.). [↑](#footnote-ref-2)
